# Chemical Optimization of Selective *Pseudomonas aeruginosa* LasB Elastase Inhibitors and Their Impact on LasB-Mediated Activation of IL-1β in Cellular and Animal Infection Models

**DOI:** 10.1021/acsinfecdis.2c00418

**Published:** 2023-01-20

**Authors:** Martin J. Everett, David T. Davies, Simon Leiris, Nicolas Sprynski, Agustina Llanos, Jérôme M. Castandet, Clarisse Lozano, Christopher N. LaRock, Doris L. LaRock, Giuseppina Corsica, Jean-Denis Docquier, Thomas D. Pallin, Andrew Cridland, Toby Blench, Magdalena Zalacain, Marc Lemonnier

**Affiliations:** † Antabio SAS, Biostep, 436 rue Pierre et Marie Curie, 31670 Labège, France; ‡ Department of Microbiology and Immunology, Rollins Research Center, 1510 Clifton Rd, Atlanta, Georgia 30322, United States; § Dipartimento di Biotecnologie Mediche, Università degli Studi di Siena, Viale Bracci 16, 53100 Siena, Italy; ∥ Centre d’Ingénierie des Protéines - InBioS, University of Liège, Allée du six Août 11, 4000 Liège, Belgium; ⊥ Charles River Laboratories, 8-9 The Spire Green Centre, Harlow, Essex CM19 5TR, U.K.

**Keywords:** Pseudomonas aeruginosa, LasB, elastase, pseudolysin, antivirulence, IL-1β

## Abstract

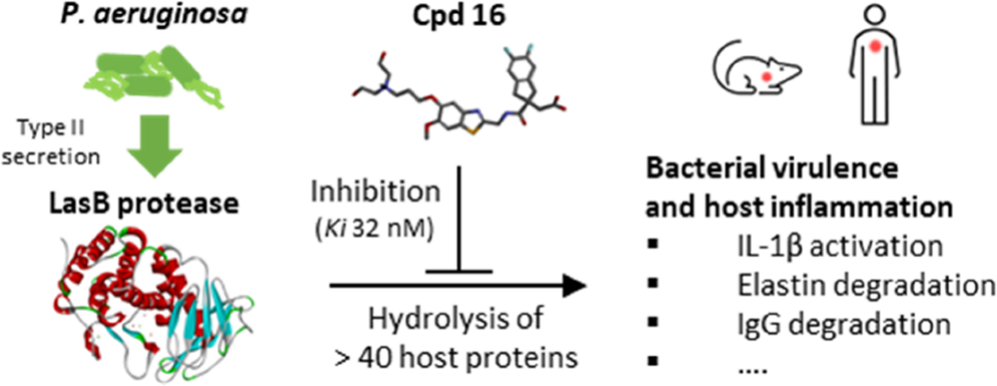

LasB elastase is a broad-spectrum exoprotease and a key virulence factor of *Pseudomonas aeruginosa*, a major pathogen causing lung damage and inflammation in acute and chronic respiratory infections. Here, we describe the chemical optimization of specific LasB inhibitors with druglike properties and investigate their impact in cellular and animal models of *P. aeruginosa* infection. Competitive inhibition of LasB was demonstrated through structural and kinetic studies. *In vitro* LasB inhibition was confirmed with respect to several host target proteins, namely, elastin, IgG, and pro-IL-1β. Furthermore, inhibition of LasB-mediated IL-1β activation was demonstrated in macrophage and mouse lung infection models. In mice, intravenous administration of inhibitors also resulted in reduced bacterial numbers at 24 h. These highly potent, selective, and soluble LasB inhibitors constitute valuable tools to study the proinflammatory impact of LasB in *P. aeruginosa* infections and, most importantly, show clear potential for the clinical development of a novel therapy for life-threatening respiratory infections caused by this opportunistic pathogen.

*Pseudomonas aeruginosa* is an opportunistic bacterial pathogen present in many natural environments that can cause fatal and debilitating diseases in humans, especially in patients whose immune responses are compromised and who are unable to clear an initial infection. Due to the large size and plasticity of its genome, *P. aeruginosa* is able to adapt to many situations and survive both the host immune response and antibiotic challenges.^1^ In fact, there are few antibiotics that can effectively and reliably kill *P. aeruginosa* because of phenotypic antibiotic tolerance, including the ability to form biofilms, and the spread of genotypically resistant strains.^1^ Patients with chronic respiratory conditions, such as bronchiectasis, chronic obstructive pulmonary disease (COPD), or cystic fibrosis (CF), are particularly vulnerable to *P. aeruginosa* infection.^2−4^ In acute pneumonia, *P. aeruginosa* infection is a major cause of acute lung injury (ALI) and acute respiratory distress syndrome (ARDS).^5,6^ More recently, increased nasopharyngeal carriage of *P. aeruginosa* and an increased incidence of *P. aeruginosa* pneumonia have been identified as consequences of SARS-COV-2 infection.^7,8^

There is an urgent need for novel therapeutic approaches to address the dual problem of antimicrobial resistance (AMR) and the lack of new antibiotics to replace those which have lost their effectiveness. One such approach is to develop drugs targeting pathogen-specific virulence factors that contribute to disease pathogenicity and/or promote pathogen colonization, rather than killing the pathogen directly, as is the case with traditional antibiotics. In *P. aeruginosa*, many virulence determinants have been identified that are essential for the establishment or maintenance of infection, including extracellular proteases, toxins, type-3 secretion, cell envelope components, and factors involved in the quorum sensing regulatory network that controls expression of many virulence determinants.^9^

One of the most promising targets for therapeutic intervention is *P. aeruginosa* elastase B (also known as pseudolysin), encoded by the *lasB* gene, whose potential as a drug target has been reviewed by Everett and Davies.^10^ The *lasB* gene is strongly conserved among *P. aeruginosa* environmental and clinical strains, being present in >98% of all genomes submitted to the Pseudomonas genome database (https://pseudomonas.com).^11^ Expression of the LasB protein is under the transcriptional control of the LasR quorum sensing regulator and, under standard laboratory growth conditions, is the most abundant protein in the secretome.^12^ The mature LasB protein is a 33 kDa metalloprotease, which is secreted from *P. aeruginosa* via the type II secretion system.^13^ LasB has a broad substrate specificity and has been shown to hydrolyze a large number and variety of human proteins, including structural components (elastin, collagen), surfactants, mucin, immunoglobulins, cytokines, antimicrobial peptides, and many others.^10^ LasB is thought to play an important role in the early stages of *P. aeruginosa* infection through degradation of host tissues and components of the innate immune response.^10,14,15^ LasB has also been shown to contribute directly to the inflammatory response through proteolytic activation of interleukin-1β (IL-1β), which is a driver of pathological inflammation.^16^

In cellular and animal studies, LasB has been shown to be cytotoxic, cause tissue damage, and impair repair processes, whereas ablation of LasB activity through *lasB* disruption or compound-mediated inhibition has been shown to result in reduced virulence^14,15,17,18^ and a lower rate of chronic lung colonization^19^ in animal infection models. In mice, secretion of LasB by clinical *P. aeruginosa* isolates has been shown to induce hemorrhagic diffuse alveolar damage (DAD).^5^ A recent study identified LasB activity in 75% of respiratory isolates from 238 intensive care unit (ICU) patients and that high levels of LasB activity were associated with increased 30-day mortality.^20^ Thus, there is strong evidence that inhibiting LasB could facilitate pathogen clearance as well as reduce disease pathology.

Several groups have investigated LasB as a potential drug target, as it is secreted outside of the bacterial cell and thus inhibitors do not need to cross two bacterial membranes to exert their action.^10^ The natural product phosphoramidon was identified as a potent inhibitor of LasB but also of many mammalian metalloenzymes,^21^ and thus unsuitable for development. Several synthetic inhibitors with thiol^22,23^ or hydroxamate^24,25^ warheads, which intercalate with the active site Zinc, have also been investigated but development has been limited by modest activity, lack of selectivity, or poor physicochemical properties.^10^ No group has yet demonstrated efficacy of a specific LasB inhibitor in a vertebrate animal infection model. We have previously described the virtual screening and exploratory chemistry leading to the identification of a novel and tractable series of indanyl carboxylate compounds with selective activity against LasB and for which binding to the LasB active site was confirmed by X-ray co-crystallography.^26^ This article describes the subsequent optimization of this series with the aim of improving potency and solubility while retaining selectivity against host metalloenzymes. We herein describe the discovery of two potent, selective compounds with high aqueous solubility and promising drug properties, representing different chemical subseries, and their use as chemical probes to investigate the impact of inhibiting LasB activity within cellular and *in vivo* models of *P. aeruginosa* infection.

## Results

### Optimization of the Indane Carboxylate LasB Inhibitor Series

The indane carboxylate **1** (originally referenced as compound 29 in the publication by Leiris et al.^26^) was confirmed as having submicromolar potency against LasB (IC_50_ 0.41 μM) and modest aqueous solubility of 0.58 mg/mL (Table 1). This was chosen as the starting point for a medicinal chemistry-driven lead optimization program aiming to improve potency and solubility whilst retaining selectivity against host metalloenzymes. All synthesized compounds were initially assessed in a fluorimetric assay measuring hydrolysis of a fluorogenic substrate Abz-Ala-Gly-Leu-Ala-4-nitrobenzylamide (Abz-AGLA-Nba) by purified LasB and IC_50_ values determined (Tables 1 and 2). Inhibition of elastin hydrolytic activity was confirmed using an orthogonal assay measuring hydrolysis of Elastin Congo Red (ECR) complex by LasB prepared directly from a *P. aeruginosa* culture supernatant (Tables 1 and 2). Inhibition of angiotensin-converting enzyme (ACE) was determined for all compounds as a primary selectivity filter for metalloenzyme specificity; no compounds showed any inhibition (ACE IC_50_ > 200 μM). Aqueous solubility was determined for most compounds (Tables 1 and 2).

**Table 1:**
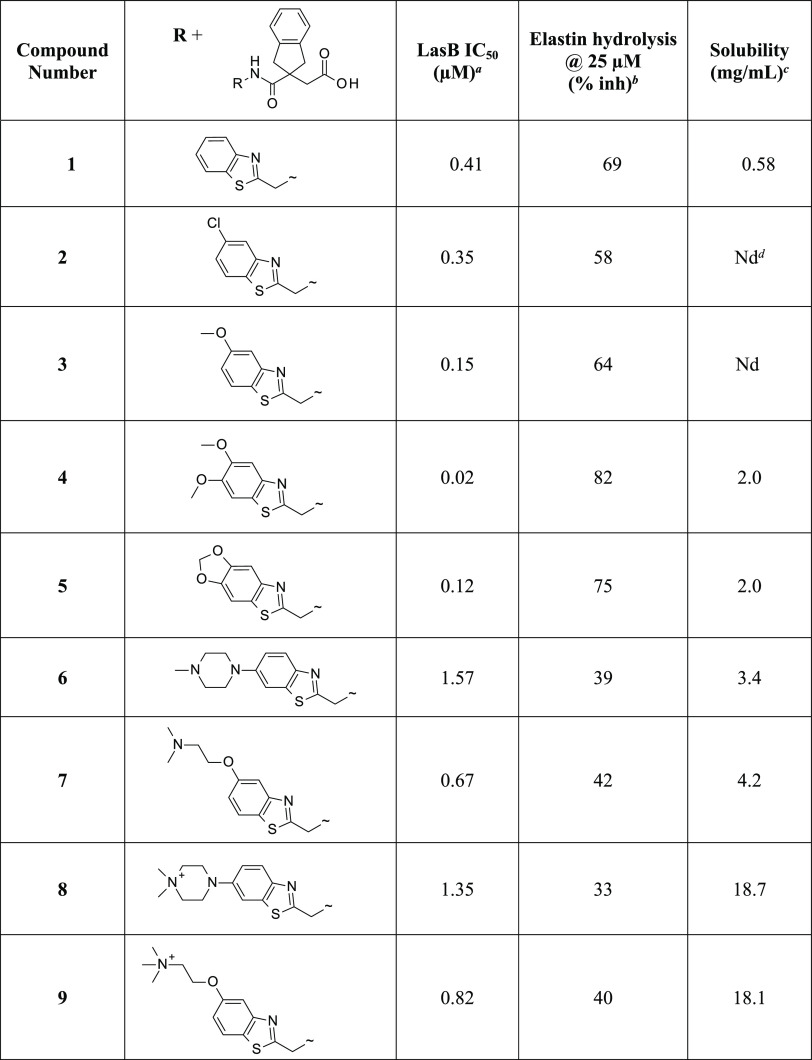
LasB Inhibitory Activities and Solubility of Substituted Benzothiazoles

a Inhibition of purified LasB enzyme in Abz assay.

b Inhibition at 25 μM inhibitor of elastin hydrolysis by dialyzed PAO1 culture supernatant in ECR assay.

c Thermodynamic solubility in PBS (pH 7.4), measuring concentration of a saturated solution of compound at equilibrium.

d not determined.

**Table 2:**
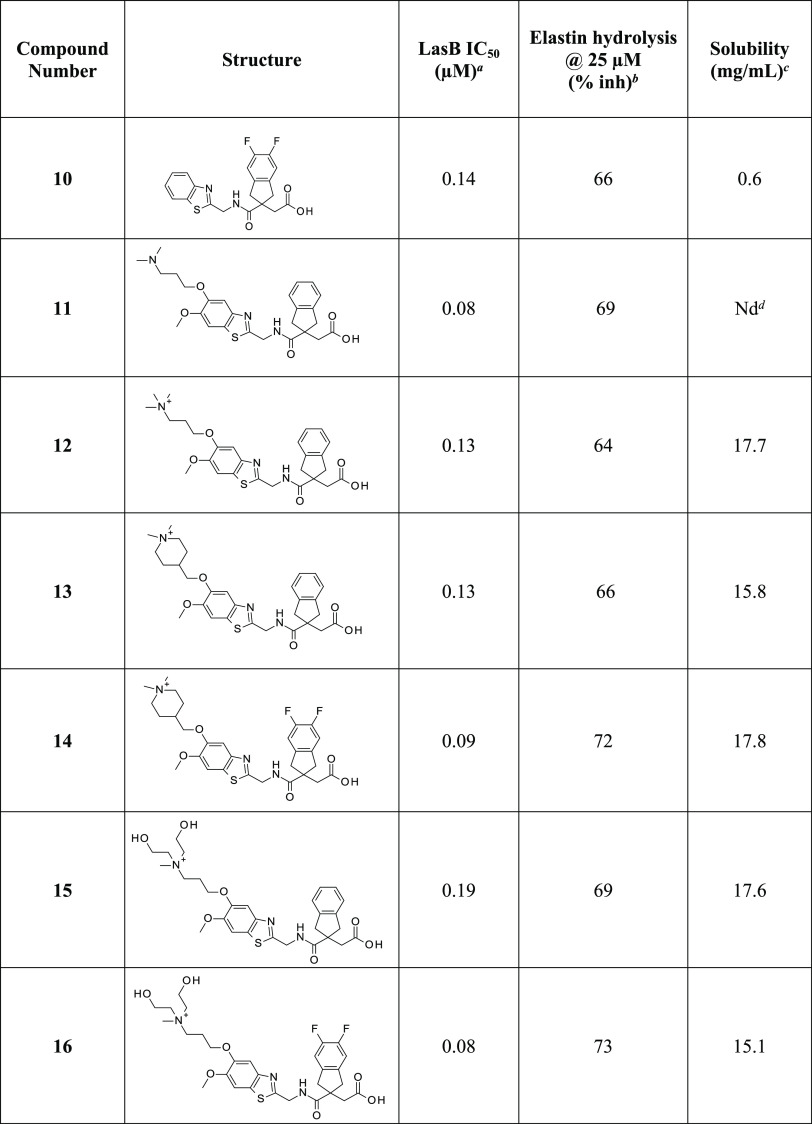
LasB Inhibitory Activities and Solubility of Substituted and Quaternized Indane Analogues

a See notes to Table 1.

As part of the initial hit expansion, various alternative heterocycles had been synthesized, but no improvements in enzyme inhibition were seen with any of them.^26^ The length of the linker between the amide carbonyl and the heterocycle had also been investigated and a single methylene, as in compound **1**, found to be optimal. In this study, substitution on the benzothiazole ring of **1** was investigated (Table 1). For example, a chloro analogue **2** was well tolerated whilst improvements in activity were seen with electron-donating oxygen substituents as in **3**, **4**, and **5**. However, although these compounds have promising LasB inhibitory activity and improved aqueous solubility (2 mg/mL), this simple series was not progressed since greater solubility was considered necessary for further development. To enhance the solubility of the compounds, a range of more complex substituents bearing solubilizing amino substituents were synthesized, e.g., the tertiary amines **6** and **7**. In general, this approach did improve the solubility over the parent analogues but with a slight reduction in activity. Quaternizing these tertiary amines to give the corresponding quaternary ammonium salts, thereby introducing a permanent positive charge, yielded **8** and **9**, which were an order of magnitude more soluble without loss of LasB inhibitory activity.

Separately, derivatization on the benzene ring of the indane generated a new subseries of difluoroindanyl analogues, such as compound **10** (Table 2). Note that disubstitution maintains the plane of symmetry, whereas a monosubstituted compound would have generated a chiral center at the quaternary carbon, introducing complications due to the existence of enantiomers. Both subseries were pursued in parallel and the final stages of lead optimization involved bringing together the improved activity of the two alkoxy substituents, as in **11**, with quaternary ammonium substituents, leading to compounds such as **12**, **13**, and **14**. Introducing hydroxy substituents into the quaternary group itself culminated in **15** and **16**, which showed both high levels of activity and solubility.

Compounds **12** and **16**, representative of the two most promising subseries, were selected for further characterization, including additional selectivity and cytotoxicity studies (Table 3). Neither compound showed inhibition of ACE, human matrix metalloproteases (MMP) 1, 2, 9, or 13. Furthermore, as expected, neither compound showed inhibition of the human neutrophil elastase (HNE), which is a serine-protease enzyme. Regarding potential cytotoxicity of the compounds, no growth inhibition of human cell lines of hepatocytes (HepG2), bronchial smooth muscle cells (BSMC), and small airway epithelial cells (SAEC) was observed with either compound at concentrations up to 100 μM. Both compounds had moderate plasma protein binding (PPB) in mice (76 and 82%, respectively) (Table 3).

**Table 3:** Selectivity, Cytotoxicity, and Plasma Protein Binding of Substituted Benzothiazoles **12** and **16**

property		compound **12**	compound **16**
selectivity (IC_50_, μM)^a^	ACE	>200	>200
	MMP-1, MMP-13	>100	>100
	MMP-2, MMP-9	>200	>200
	HNE	>200	>200
cytotoxicity (IC_50_, μM)^a^	HepG2	>100	>100
	BSMC	>100	>100
	SAEC	>100	>100
plasma protein binding (10 μM)	PPB (mouse) % bound	76	82

a Inhibition of ACE (rabbit angiotensin-converting enzyme), selected MMPs (human matrix metalloproteases), HNE (human neutrophil elastase), HepG2 (hepatocyte cell line), BSMC (bronchial smooth muscle cells), and SAEC (small airway epithelial cells).

### Interaction with LasB

**Figure 1:**
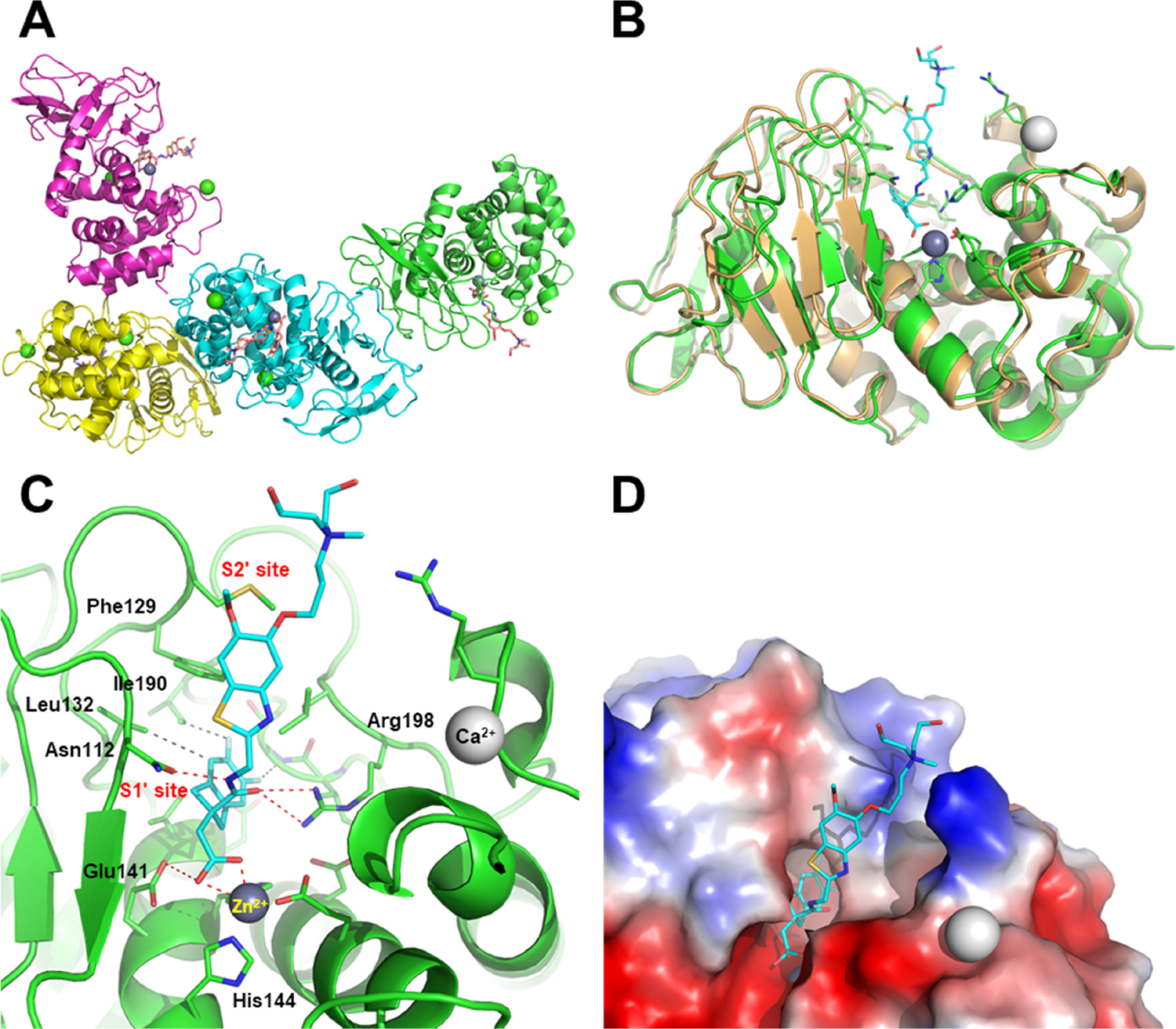
Crystal structure of *P. aeruginosa* LasB inhibited by compound **16** (PDB code, 7QH1). (A) Content of the asymmetric unit showing the presence of four subunits, all containing an inhibitor molecule in their active site (all four subunits were nearly identical, r.m.s.d. 0.10–0.20 Å); (B) superimposition of native LasB (PDB code, 1EZM; orange) with subunit A of the LasB:**16** complex (green; r.m.s.d., 1.16 Å); (C) close-up view of LasB (green) showing the interaction of compound **16** (cyan) with several key residues and the catalytic zinc cofactor (gray sphere) (see text for details); and (D) surface representation of the LasB active site showing the close contacts with the difluoroindanyl and benzothiazole moieties of the inhibitor and the protruding, poorly interacting, quaternary ammonium substituent.

A crystal structure of the LasB:**16** complex was obtained at a maximum resolution of 2.74 Å (Figure 1, see the Supporting Information for methodology, data collection, and refinement statistics). As anticipated, and observed previously for compound **1**,^26^ compound **16** was found to interact with Zn^2+^ in the active site of LasB through its carboxylic acid functionality, with its indane moiety sitting in the S1′ lipophilic pocket. Stabilizing interactions were found between compound **16** and certain LasB residues, namely (i) between the oxygen and nitrogen atoms of the amide, forming a strong bidentate interaction to Arg198 and Asn112, respectively, (ii) the benzothiazole sitting in a groove adjacent to the S2′ pocket, stabilized by pi-stacking interaction to Phe129, and (iii) the nitrogen atom of the benzothiazole ring which forms part of a water-mediated network extending to the base of the shallow S2′ pocket.

### Mechanism of LasB Inhibition

The mechanism of LasB inhibition was investigated for compounds **12** and **16** by measuring the rate of Abz-AGLA-Nba hydrolysis at different substrate and inhibitor concentrations. Dixon plot analysis (1/v against [I]) for both compounds was consistent with a competitive inhibition model (Figure S1 in the Supporting Information). The competitive inhibition constants (*K*_*i*_), determined from the point of intersection, were 35 and 32 nM for **12** and **16**, respectively. This mechanism of inhibition was further supported by observations from structural X-ray crystallographic studies with **16**, which showed the inhibitor interacting with the S1′ substrate binding pocket within the LasB active site.

### Inhibition of LasB Variants from *P. aeruginosa* Clinical Isolates

Compounds **12** and **16** were discovered as a result of optimizing potency against the LasB protein expressed from the wild-type (WT) reference strain *P. aeruginosa* PAO1. To understand the relative prevalence of the WT LasB protein versus other variants in clinical respiratory *P. aeruginosa* strains, the full-length *lasB* gene was amplified by PCR from 255 clinical isolates (obtained from CF sputum samples). Sequence analysis of the PCR products indicated that 120 strains (47%) had identical sequences to PAO1 whilst the remainder revealed variations resulting in one to five amino acid substitutions in their putative *lasB* gene product. The two most common variants contained either a single substitution, S241G (*n* = 70, 28%), or five substitutions, Q102R, S241G, D244N, K282N, and R471S (*n* = 51, 20%); hence, the three major *lasB* genotypes (including the PAO1 WT) accounted for 95% of *lasB* gene sequences. Several minor variants were also identified and are listed in Table 4. Most changes were identified in the mature peptide, however some occurred in the propeptide domain, which could affect LasB processing (marked in italics in Table 4).

**Table 4:** Relative Proportion, Specific Activity of LasB Genotypic Variants Identified in 255 *P. aeruginosa* CF Sputum Isolates and Inhibition by Compounds **12** and **16**

LasB genotype^a^	number (%)	LasB spec. act^b^	12 IC_50_, nM^c^	16 IC_50_, nM^c^
WT (PAO1 reference)	120 (47%)	6.5	222	177
S241G	70 (28%)	6.3	221	206
*Q102R*, S241G, D244N, K282N, R471S	51 (20%)	6.3	183	178
T65I	3 (1%)	5.4	216	182
*Q71L*	3 (1%)	3.3	214	177
M325V	1 (<1%)	d		
S460T	5 (2%)	6.9	211	201
S241G, A497S	1 (<1%)	d		
*N68S*, *T120I*, *A122T*, S241G, S436L	1 (<1%)	1.6	267	253

a Italics refer to amino acid substitutions occurring in the LasB propeptide domain (aa 24–198).

b Mean specific activity (RFU/min/mg protein × 10^8^) measured using the Abz-AGLA-Nba substrate and normalized to mg LasB protein (Figure S2), determined from two independent supernatant samples (unless only one strain identified).

c Mean of IC_50_ values from each supernatant sample.

d Supernatants with active LasB not generated.

Culture supernatants from representative strains, including duplicate preparations of the three major genotypes, were prepared and demonstrated similar LasB protein production as determined by Western blotting (Supporting Information, Figure S2) and specific activities with regard to hydrolysis of the Abz-AGLA-Nba peptide substrate (Table 4). For all variants tested, LasB activity was sensitive to inhibition by both compounds **12** and **16** to a similar extent, regardless of sequence variation, with **16** appearing to be slightly more potent than **12** against all variants. Querying the Pseudomonas Genome Database (https://pseudomonas.com) comprising 4955 *P. aeruginosa* genome entries (at the time of writing), including environmental, human and veterinary isolates, revealed the same three common genotypes were present in 87% of deposited *lasB* sequences. Taken together, these data indicate a high degree of *lasB* gene sequence conservation and that compounds **12** and **16** are equipotent against the most common LasB variants, including those found in clinical CF isolates.

### Inhibition of LasB-Mediated Proteolysis of Host Substrates

#### IgG

**Figure 2:**
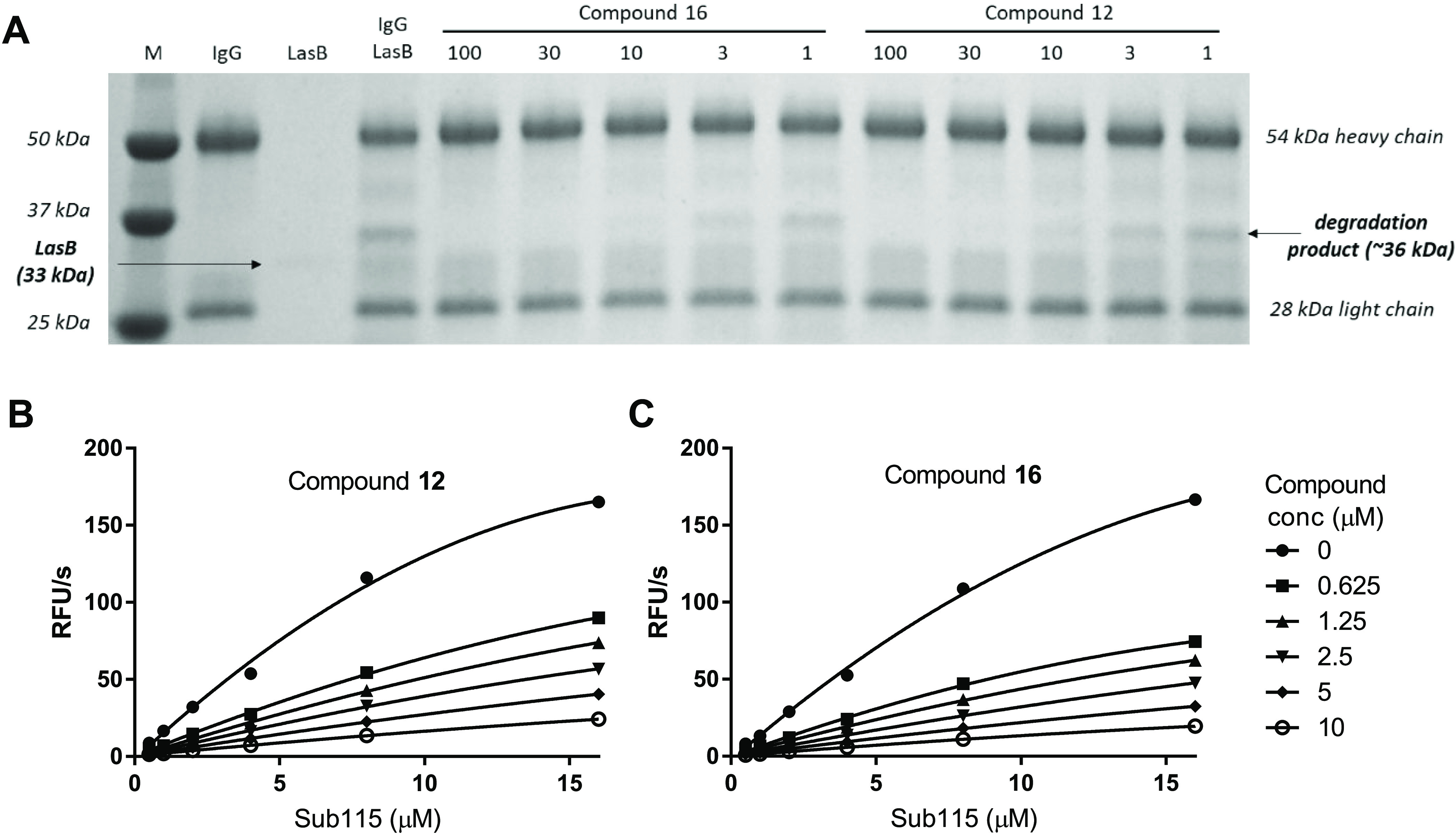
Inhibition of LasB-mediated proteolysis of host substrates by compounds **12** and 1**6**. (A) Proteolysis of IgG by LasB results in the generation of a 36 kDa degradation product, which is inhibited by increasing concentrations of LasB inhibitors **12** and **16**. (B, C). Hydrolysis of different concentrations of the pro-IL-1β fluorescent peptide Sub115 by LasB was inhibited by compounds **12** (B) and **16** (C). Experiments were performed twice on two separate occasions (results from a single set of experiments are shown).

LasB has been shown to degrade and inactivate IgG both *in vitro* and *in vivo*.^27^ To confirm if compounds **12** and **16** were able to inhibit this activity, human serum IgG was incubated with purified LasB in the presence of varying concentrations of inhibitors and the products analyzed by SDS-PAGE. The generation of a 37 kDa degradation product by LasB was clearly inhibited by increasing concentrations of **12** and **16** in a dose-responsive manner, with complete inhibition observed at 10 μM (Figure 2A). Quantification of band intensity allowed the % inhibition to be determined for each inhibitor concentration and IC_50_ values to be calculated as 2.59 and 1.29 μM for compounds **12** and **16**, respectively.

#### IL-1β

Previous studies have shown that LasB cleaves the N-terminal domain of pro-IL-1β, thereby generating a mature active form of IL-1β in a manner analogous to the normal host activation mechanism involving cleavage by caspases.^16^ This activity can be followed spectrophotometrically via hydrolysis of an internally quenched fluorescent peptide Sub115 (McA-HDAPVRSLN-K-Dnp), which incorporates the LasB cleavage site from pro-IL-1β. To determine whether compounds **12** and **16** were able to inhibit this activity, Sub115 hydrolysis was measured in the presence of varying concentrations of substrate and inhibitors (Figure 2B). Compounds **12** and **16** both inhibited cleavage of the peptide in a dose-responsive manner with mean *K*_*i*_ values from two independent sets of experiments of 0.704 μM (SD 0.113) and 0.475 μM (SD 0.016), respectively. These values, although still submicromolar, were higher than those determined with the Abz-AGLA-Nba hydrolysis assay, perhaps due to the different ion composition of the respective assay buffers.

**Figure 3:**
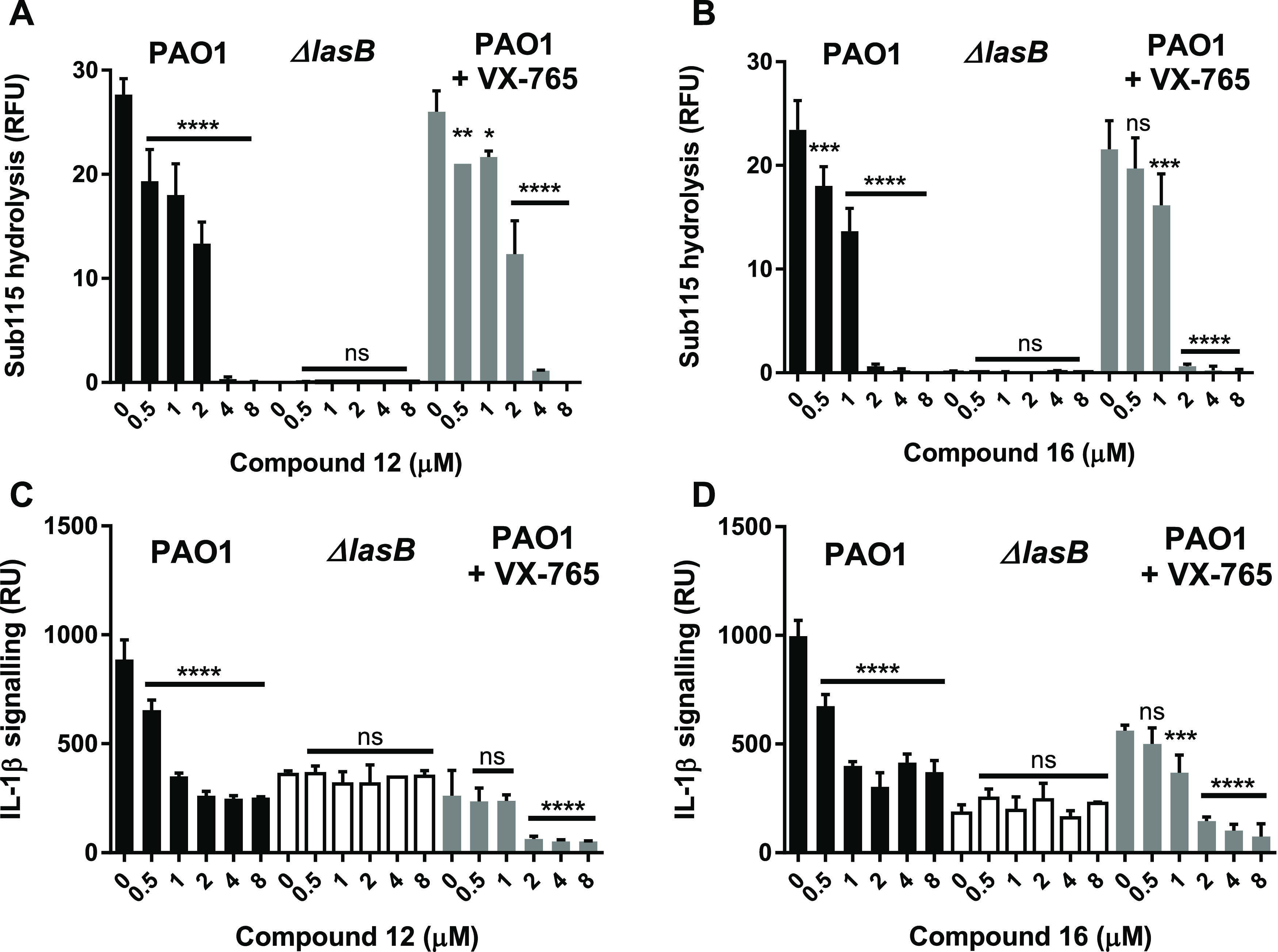
Compounds **12** and **16** inhibit secreted LasB and activation of IL-1β in *P. aeruginosa*-infected THP-1 macrophages. Compounds **12** and **16** at concentrations from 0.5 to 8 μM were added to THP-1 macrophages immediately prior to infection (MOI 10:1) with *P. aeruginosa* PAO1 (black), PAO1Δ*lasB* (white), or PAO1 in the presence of the caspase inhibitor VX-765 (5 μM) (gray). Controls with no compounds (0 μM) were included for each condition. Culture supernatants were collected after 2 h for analysis. Top panel shows the effect of compounds **12** (A) and **16** (B) on LasB activity as measured by hydrolysis of the N-terminal pro-IL-1β fluorescent peptide (Sub115). Bottom panel shows the effect of compounds **12** (C) and **16** (D) on activation of cytokine IL-1β as measured by induction of a signal in an IL-1 luciferase cell reporter assay. Statistical significance was determined by ANOVA with a Dunnett post-test: * *p* < 0.05; ** *p* < 0.01; *** *p* < 0.001; **** *p* < 0.0001; ns, not significant.

### Effects on LasB-Mediated IL-1β Activation in *P. aeruginosa*-Infected Macrophages

Further to showing *in vitro* inhibition of LasB-mediated pro-IL-1β proteolysis, experiments were performed to determine the effect of compounds **12** and **16** on IL-1β activation and functionality in human macrophage cell cultures infected with *P. aeruginosa* PAO1, or an isogenic *lasB* deletion mutant thereof (Δ*lasB*). In these experiments, the levels of LasB activity in culture supernatants were measured via hydrolysis of the Sub115 fluorescent peptide, while the effect of LasB activity on IL-1β activation (functionality) was measured using a luciferase reporter cell line sensitive to IL-1β induction (Figure 3). Infection of THP-1 macrophages with *P. aeruginosa* PAO1 (but not with Δ*lasB*) resulted in high levels of LasB activity in the supernatant, as measured by hydrolysis of Sub115, and correspondingly high levels of activated IL-1β, as measured by generation of a robust signal in the IL-1 reporter assay. Inclusion of increasing concentrations of either inhibitor resulted in statistically significant (*p* < 0.0001) reductions in LasB activity, with complete abolition of activity observed at 4 μM. Concordant with this, both inhibitors also resulted in similarly significant (*p* < 0.0001) decreases in levels of IL-1β activation (EC_50_ ∼ 0.5 μM); however, there remained a background level of IL-1 signal induction in the reporter assay even at the highest concentrations of **12** or **16**, indicating a level of residual IL-1β activation independent of LasB activity. Compound **16** was more potent in inhibiting pro-IL-1β peptide (Sub115) hydrolysis by *P. aeruginosa*-infected THP-1 culture supernatants, consistent with that observed in the *in vitro* experiments; however, inhibition of IL-1β activation appeared similar between the two compounds.

The contribution of host pathways to IL-1β activation was investigated using the caspase-1 inhibitor VX-765. When added to infected THP-1 cells, VX-765 (5 μM) had no effect on LasB activity (as expected) but resulted in reduced IL-1β activation. Co-administration of both VX-765 and LasB inhibitor resulted in an additive effect, resulting in near total ablation of the IL-1 signal. These results indicate that IL-1β can be activated independently by LasB and/or caspase-1 (the latter acting through the canonical inflammasome activation pathway) and that these activities can be specifically inhibited by LasB inhibition (**12** or **16**) and caspase inhibition (VX-765), respectively.

### Pharmacokinetic (PK) Properties

**Figure 4:**
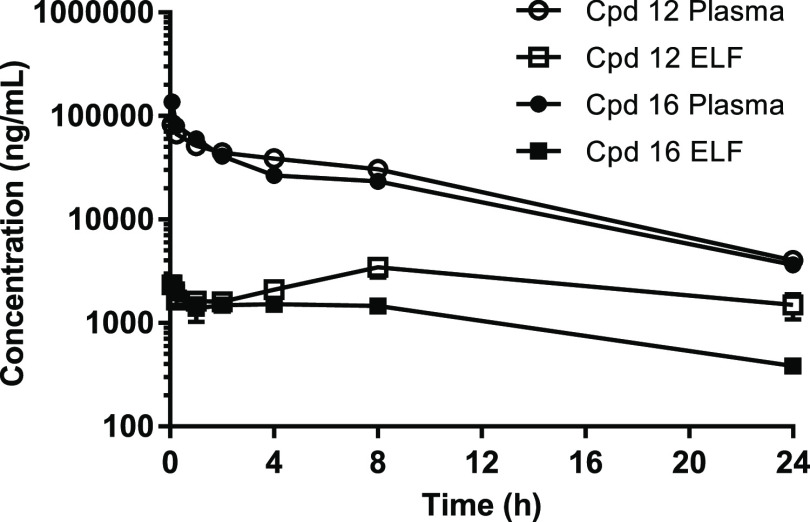
Plasma and ELF PK in mouse after single 10 mg/kg IV dose of compounds **12** and **16**. Epithelial lining fluid (ELF) concentrations were determined by normalizing bronchoalveolar lavage fluid (BALF) concentrations to plasma urea concentrations to adjust for dilution during the sampling procedure.

The PK parameters of compounds **12** and **16** were investigated in both plasma and lung epithelial lining fluid (ELF) after intravenous (IV) administration of compounds at 10 mg/kg in mice (Figure 4 and Table 5). The results were similar for both compounds, showing good plasma exposure (610.4 and 517.1 μg*h/mL, respectively) and half-life (∼6 h), and ELF concentrations (*C*_0_) over 100-fold the LasB *K*_*i*_ determined for the inhibitors. Accordingly, these levels of lung penetration (9.5 and 5.2%, respectively) warranted the evaluation of the compounds in an animal model of lung infection using IV dosing.

**Table 5:** PK Parameters in Mouse of Substituted Benzothiazoles **12** and **16**

PK parameters after single 10 mg/kg IV dose		compound 12	compound 16
plasma	*C*_0_ (μg/mL)^a^	92.2	175.7
	AUC_last_ (μg*h/mL)^b^	610.4	517.1
	CL (mL/min/kg)^c^	0.3	0.3
	*t*_1/2_ (h)^d^	6.2	6.5
ELF^e^	C_0_ (μg/mL)	2.7	2.7
	AUC_last_ (μg*h/mL)	57.7	27.0
	CL (mL/min/kg)	1.5	5.2
	*t*_1/2_ (h)	25.8	9.5
	lung penetration (AUC_last plasma_/AUC_last ELF_)	9.54%	5.22%

a Concentration at time zero.

b Area under the curve between first and last measurement.

c Clearance.

d Half-life.

e Epithelial lining fluid; concentrations determined by normalizing bronchoalveolar lavage fluid (BALF) concentrations to plasma urea concentrations to adjust for dilution during the sampling procedure.

### *In Vivo* Efficacy in a Mouse *P. aeruginosa* Lung Infection Model

**Figure 5:**
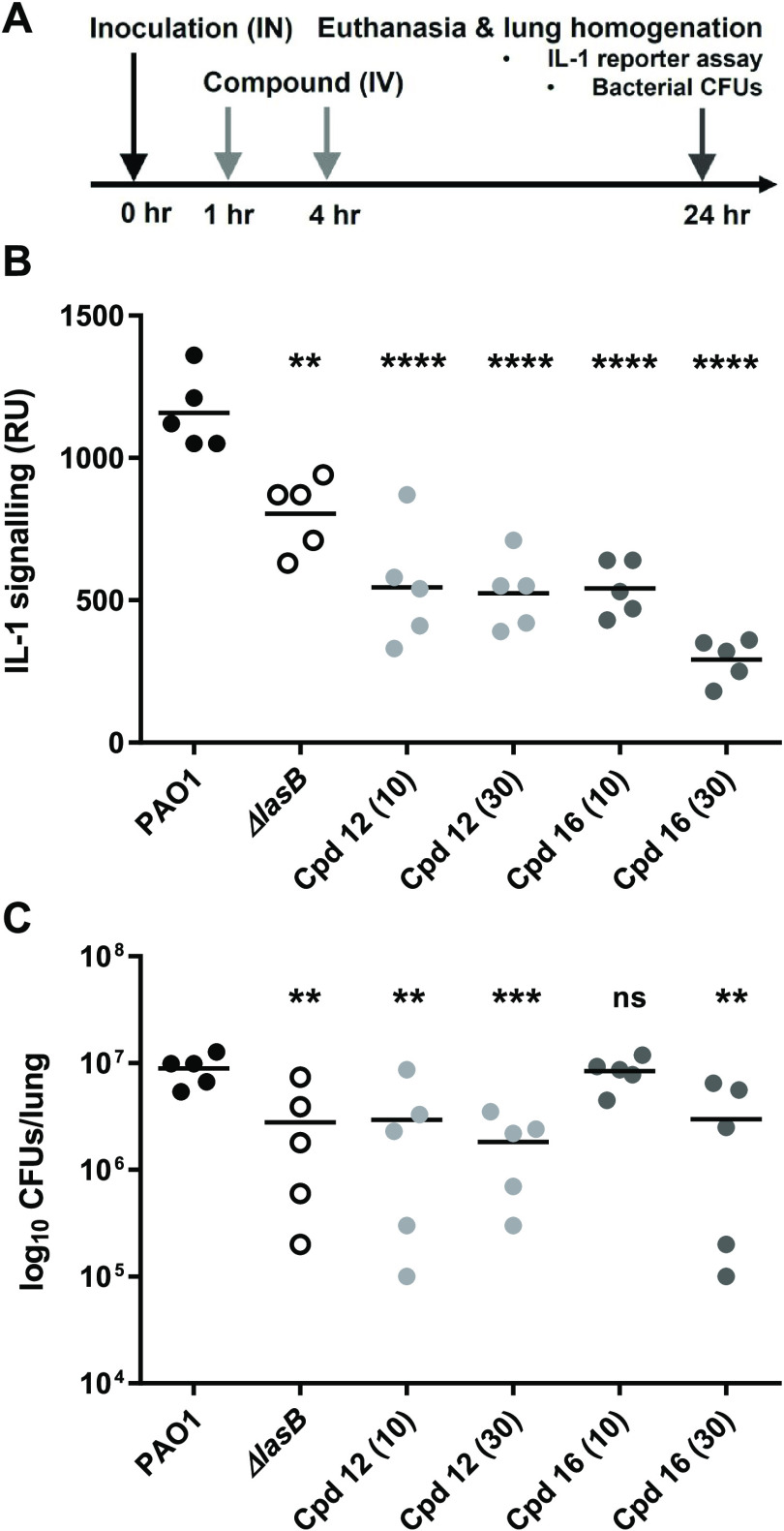
Compounds **12** and **16** inhibit LasB-mediated IL-1 β activation and reduce *P. aeruginosa* numbers in a mouse lung infection model. (A) Schematic representation of mouse infection, drug treatment, and endpoint measurements: doses of either 10 or 30 mg/kg compound were administered to the tail vein of immunocompetent C57Bl/6 mice at 1 and 4 h post-intranasal inoculation. (B) Measurement of activated IL-1β in lung homogenates at 24 h post-infection via luminescence (RU) in cell reporter. (C) Measurement of bacterial cell numbers (CFUs) in lung homogenates at 24 h post-infection. The horizontal line represents the mean value for each dataset. Statistical significance was determined by ANOVA with a Dunnett post-test: ** *p* < 0.01; *** *p* < 0.001; **** *p* < 0.0001; ns, not significant.

To investigate if compounds **12** and **16** were able to engage the LasB target *in vivo*, in the context of a *P. aeruginosa* lung infection, and to determine the consequences of that inhibition, immunocompetent C57Bl/6 mice were infected intranasally with *P. aeruginosa* PAO1 and treated with two doses of inhibitors, 10 and 30 mg/kg, administered IV at 1 and 4 h post-infection (Figure 5A). A control group of PAO1-infected animals treated with vehicle and a group of animals infected with the isogenic Δ*lasB* strain were included in the study. Mice were euthanized 24 h post-infection and their lung homogenates assayed for activated mature IL-1β (using the IL-1 reporter assay) and bacterial numbers (by CFU enumeration).

In this experiment, intranasal inoculation of mice with PAO1 induced a strong IL-1 signal and a consistent lung infection at 24 h (∼10^7^ CFUs/lung) (Figure 5B,C). In contrast, inoculation with the Δ*lasB* strain resulted in significantly lower IL-1 signal induction (*p* < 0.01) with reduced (and more variable) bacterial burden (*p* < 0.01; mean 2.8 × 10^6^ CFUs/lung; range 10^5^–10^7^ CFUs/lung), indicating reduced virulence. This shows that *P. aeruginosa* infection in an animal model induces IL-1β activation in a similar manner to that observed in the cellular model and that IL-1β activation is dependent on the capability of the pathogen to produce LasB. In addition, the data indicate that LasB contributes to the pathogenicity of *P. aeruginosa*. Treatment with the two LasB inhibitors, **12** and **16**, resulted in significantly decreased levels of activated IL-1β in lung homogenates (up to 4-fold), compared to the PAO1-infected control group (*p* < 0.0001), surpassing that observed in the Δ*lasB* group. Small but statistically significant (*p* < 0.01) log reductions in bacterial numbers (CFUs), equivalent to that observed in the Δ*lasB* group (0.51), were also observed with **12** at both doses (0.48 and 0.69) and with **16** at the higher dose (0.47).

These data clearly demonstrate that both compounds **12** and **16** dosed IV were able to penetrate the lung and inhibit the LasB enzyme secreted by the infecting *P. aeruginosa* bacterium. Furthermore, inhibition of LasB resulted in decreased activation of IL-1β and reduced virulence as evidenced by reductions in bacterial numbers at 24 h.

## Discussion

The use of antivirulence drugs to treat *P. aeruginosa* infections offers the exciting possibility of intervening in the course of infection (on the side of the patient) as an alternative or a supplement to antibiotic use. Many virulence factors have been identified, however understanding the contribution of individual determinants to bacterial pathogenicity and providing evidence for target validation is challenging. The use of genetic mutants has helped identify potential targets but does not provide information on the effect of inhibiting the active target within an infection context or control for other consequences that may result from disruption of that gene. Only by inhibiting the target activity *in situ* can its function be properly explored. For this, specific chemical probes are required with the potency, metabolic stability, and PK properties necessary to achieve inhibitory concentrations at the target site of action. In this respect, such probes share many of the properties required of a drug candidate and necessitate a major investment in chemical optimization, and hence can often only be generated as part of a drug development program.

In this study, a LasB inhibitor series was optimized to improve its potency and solubility, whilst retaining selectivity against host metalloenzymes. Modifications to the benzothiazole core yielded increased potency without losing selectivity, whilst addition of a quaternary side chain greatly improved solubility. Lead compounds **12** and **16** were shown to be potent competitive inhibitors of LasB with *K*_*i*_ values in the nanomolar range (35 and 32 nM, respectively). An X-ray crystal structure confirmed binding of **16** into the LasB active site via the indanyl moiety, while the quaternary side chain protruded outside of the active site into a solvent-exposed region. Both **12** and **16** were confirmed as selective versus other metalloenzymes (ACE, MMPs) and possessed druglike properties commensurate with their use as *in vivo* chemical tools, i.e., low cytotoxicity, metabolic stability and suitable PK (including penetration into lung fluids). *In vitro* inhibition of LasB-mediated hydrolysis of host target proteins (elastin, IgG) was also confirmed.

To investigate (i) the role of LasB within the context of a *P. aeruginosa* infection and (ii) the potential of **12** and **16** as candidates for drug development, we studied their effect in cellular and animal models of infection. Previously, it has been reported that LasB proteolytically cleaves the N-terminal peptide domain of immature IL-1β resulting in activation of IL-1β and induction of signal in an IL-1 cell reporter assay.^16^ Furthermore, loss of LasB activity through gene deletion, or inhibition by nonspecific metalloenzyme inhibitors, has been shown to suppress IL-1β activation.^16^ Consequently, IL-1β activation was used in this study as a marker to probe the effect of the two specific LasB inhibitors.

First, inhibition of LasB-mediated cleavage of the IL-1β N-terminal peptide by both **12** and **16** was confirmed *in vitro*. Next, the compounds were shown to inhibit both secreted LasB and IL-1β activation in a *P. aeruginosa*-infected THP-1 macrophage cell line. Interestingly, complete inhibition of LasB (as measured via hydrolysis of a pro-IL-1β peptide) did not result in complete ablation of the IL-1 signal, indicating a residual level of IL-1β activation in the absence of LasB activity. This residual activity was shown to be due to activation by host caspases, as evidenced by complete ablation of the IL-1 signal when both LasB inhibitor and an inhibitor of endogenous caspases (VX-765) were added together.

Finally, LasB inhibition was studied in a mouse lung *P. aeruginosa* infection model. Compounds were administered IV at doses predicted from PK analyses to result in super-inhibitory concentrations at the site of infection (i.e., the lung ELF). IL-1β activation (as evidenced by reduction in IL-1 signaling by lung fluid samples) was inhibited by both compounds in a dose-responsive manner and the burden of *P. aeruginosa* infection was also reduced at 24 h. Notably, reduction of IL-1 signal (but not reduction in bacterial numbers) was more pronounced at the highest compound dose than in the *lasB* deletion control group. This may point to unidentified off-target activities of the compounds but could also reflect the greater immunogenic potential of a *lasB* null strain (due to retention of flagella, a potent TLR agonist degraded by LasB),^28,29^ making direct comparison difficult. This underlines the importance of probing target function through specific inhibitors rather than just relying on functional genetic knockouts.

The finding that LasB activity is associated with higher IL-1β activation and bacterial numbers during *P. aeruginosa* infection is consistent with the findings from a recent study by Zupetic et al., who showed in a mouse lung infection model with clinical isolates (from ICU patients) that high LasB activity is associated with increased bacterial burden and enhanced inflammatory cytokine production, including increased IL-1β.^20^ Mean reductions in bacterial burden were modest in these experiments due to the large variability in responses within each treatment group. This is perhaps to be expected since clearance of bacteria will depend on the immune status of individual animals, rather than being due to the direct effects of the compound on bacterial viability (the compounds have no antibacterial activity). Future experiments could investigate whether improved clearance, and less variation, are observed over longer time periods and whether treatment prevents spread to other organs.

Compounds **12** and **16** represent an advance on previously described LasB inhibitors, being potent and specific for LasB (compared to mammalian metalloproteases), noncytotoxic, and, most importantly, efficacious in a vertebrate model of lung infection. Further work will probe the efficacy of these compounds, with regard to suppression of LasB-mediated lung damage and reduction of *P. aeruginosa* pathogenicity and will investigate delivery of compounds via the inhalation route, which is anticipated to deliver higher concentrations to the target site. The goal of such studies will be to demonstrate the potential therapeutic benefit of LasB inhibition and justify the continued development of the LasB inhibitors described in this manuscript.

LasB inhibitor drugs could be used as adjuncts to antibiotic therapy for treatment of acute infections; however, they may also have application as standalone drugs when antibiotic use has not been initiated, or where it is not recommended. Situations where standalone use could be beneficial include *P. aeruginosa* colonization in people with chronic respiratory infections (e.g., bronchiectasis) or prophylaxis prior to high-risk hospital procedures. Such “pathogen disarming” approaches offer the prospect of disrupting the course of the infection and enabling immune-mediated clearance while avoiding collateral damage to the host’s microbiome due to inappropriate use of antibiotics.

## Conclusions

To summarize, this study confirms that (i) LasB is expressed and is active in *P. aeruginosa*-infected cells and mouse lungs, (ii) LasB drives an increase in proinflammatory IL-1 signaling through an independent mechanism of pathogen-mediated IL-1β activation, and (iii) inhibition of secreted LasB activity *in situ* results in a reduced inflammatory signal as well as an impaired ability of the pathogen to establish a robust infection. These results underline the importance of LasB as a virulence factor in *P. aeruginosa* respiratory infections and validate the approach of developing small molecule inhibitors of LasB as a therapeutic strategy. This work also supports the further evaluation of compounds **12** and **16** as preclinical development candidates, paving the way toward a new treatment paradigm for life-threatening *P. aeruginosa* infections.

## Methods

### General Chemistry Procedures

Reactions were performed under argon or nitrogen using dried glassware and solvents. Commercially available reagents and solvents were used as supplied. Reactions were conducted at room temperature (RT) unless otherwise stated and monitored by standard thin-layer chromatography or liquid chromatography–mass spectrometry (LC–MS) techniques. Silica gel chromatography was performed with standard silica columns packed with silica gel (Merck silica gel 40–63 μm) or using commercial prepacked silica cartridges (Biotage) and eluting with solvent combinations as described. 1H spectra were recorded using 500 MHz (Bruker), 400 MHz (Bruker), 400 MHz (Varian), and 300 MHz (Varian) instruments in the deuterated solvents as indicated. See the Supporting Information for details. All final testable compounds were >95% pure as determined by NMR and LC–MS.

### Protein Production and Purification

Native LasB protein was purified from culture supernatants of *P. aeruginosa* strain PAO1 (Charles River Laboratories, U.K.). Briefly, the sample was concentrated by ultrafiltration and the protein was purified by chromatography using an anion exchange (pH 8.5) step followed by gel filtration. This procedure yielded an enzyme preparation (0.8 mg/mL) of >98% purity. The enzyme was stored at −80 °C.

### Protein Crystallization and X-ray Diffraction Data Collection

Purified LasB was co-crystallized with compound **16** in hanging drops at 20 °C, comprising 1 μL of ligand solution (1 mM in 0.1 M MOPS pH 6.5, 1.3–1.8 M NH_4_SO_4_) with 1 μL of LasB protein (10 mg/mL). Diffraction data were obtained at beamline ID30A-1 (ESRF, Grenoble, France), and data were processed using standard methods (molecular replacement was performed using PDB code 1EZM as the template^30^). See the Supporting Information for details.

### LasB Inhibition Assays

LasB inhibition assays were performed using purified LasB enzyme (1 ng/well) for compound screening or using culture supernatants (prepared as described in the ECR assay) for testing LasB variants. Hydrolysis of the fluorogenic peptide substrate 2-aminobenzoyl-Ala-Gly-Leu-Ala-4-nitrobenzylamide (Abz-AGLA-Nba; Peptide International) in 50 mM Tris-HCl (pH 7.4), supplemented with 2.5 mM CaCl_2_ was followed using an Envision microplate reader (PerkinElmer) at 315 nm excitation and 430 nm emission. For screening of lead compounds, assays were performed in the presence of 0.01% Triton X-100 with a range of inhibitor concentrations from 0.003 to 3.2 μM. IC_50_ values were determined from Hill plots using Dotmatics Studies ELN software. For the mechanism of inhibition studies, hydrolysis rates were measured in the presence of variable concentrations of substrate and inhibitor, and the inhibition constant (*K*_*i*_) was determined using Dixon plot analysis.^31^

Inhibition of elastin hydrolysis by native LasB, secreted in *P. aeruginosa* culture supernatants, was measured using the Elastin Congo Red (ECR) assay. Culture supernatants from *P. aeruginosa* strain PAO1, grown in LB medium at 37 °C for 24 h, were filtered and dialyzed against 50 mM Tris-HCl pH 7.4, 2.5 mM CaCl2 solution at 4 °C and supplemented with 0.01%Triton X-100, 10 mg/mL ECR (Sigma) and 25 μM inhibitor compounds. The reaction mixture was incubated for 16 h at 37 °C with shaking before centrifugation and recovery of the supernatant and measurement of solubilized congo red by absorbance (OD_495_) using an EnSight multimode plate reader (PerkinElmer).

### Specific Activity of LasB Variants

For determination of specific activities of LasB variants, LasB activity in culture supernatants was measured by hydrolysis of Abz-AGLA-Nba (as described above) and normalized to mg LasB protein, as estimated from SDS-PAGE Western blots probed with anti-LasB polyclonal antibody (see Figure S2).

### Selectivity Assays

Selectivity of compound inhibition was evaluated against other proteases at concentrations between 0.4 and 200 μM. Activity against rabbit angiotensin-converting enzyme (ACE, Sigma) was assessed using 10 μM fluorogenic substrate Abz-Phe-Arg-Lys(Dnp)-P (Enzo Life Sciences) in 100 mM Tris-HCl pH 7, 50 mM NaCl, 10 μM Zn SO_4_. Activity against human matrix metalloproteases (MMP)-2 and MMP-9 was assessed in 50 mM HEPES pH 7.5, 10 mM CaCl_2_, 0.01% Triton X-100 using 4 μM fluorogenic substrate Mca-Pro-Leu-Gly-Leu-Dpa-Ala-Arg-NH_2_ (Enzo Life Sciences). Activity against MMP-1 and MMP-13 was assessed in 25 mM HEPES pH 7.5, 100 mM NaCl, 100 mM CaCl_2_, 0.0005% Brij using 7.5 μM fluorogenic substrate Mca-Lys-Pro-Leu-Gly-Leu-Dpa-Ala-Arg-NH_2_ (Sigma) for MMP-1 and 1.25 μM fluorogenic substrate Mca-Pro-Cha-Gly-Nva-His-Ala-Dpa-NH_2_ (Millipore) for MMP-13. Activity against human neutrophil elastase (HNE, Enzo Life Sciences) was assessed in 100 mM HEPES pH 7.5, 500 mM NaCl, 0.01% of Triton X-100 using 100 μM fluorogenic substrate MeOSuc-AAPV-AMC (Enzo Life Sciences).

### Cytotoxicity Screening

Bronchial smooth muscle cells (BSMC, Lonza CC-2576), small airway epithelial cells (SAEC, Lonza CC-2547), or HepG2 cells (ATCC HB-8065) were seeded in 96-well plates at a density of 25,000 per well. Culture of these cells throughout the protocol utilized incubation at 37 °C, 5% CO_2_ in a humidified atmosphere, with BSMC and SAEC cells cultured in cell-specific media (Lonza) and HepG2 cells cultured in RPMI 1640 medium containing Glutamax (Gibco) and 10% fetal bovine serum. Plates containing BSMC cells were collagen coated to assist with cell adhesion. Following an overnight settling period, media was replaced with fresh media, with, or without, compound. Compound concentrations tested were 1, 3, 10, 30, and 100 μM, in duplicate. The cells were incubated with compound for 24 h. Control (1% DMSO) and positive standards terfenadine- and salmeterol-treated groups were included for both cell lines. Following the 24 h compound incubation period, 10 μL of MTS reagent (Promega G5421) was added to 50 μL cell media and adhered cells, and the plates were incubated for 45 min at 37 °C, 5% CO_2_ in a humified atmosphere. The reaction was stopped with 12.5 μL of 10% SDS, plates briefly shaken, and absorbance (490 nm) read using a Spectramax M5e plate reader (Molecular Devices). Percentages of viable cells were calculated as: (test sample Abs 490 nM-mean blank Abs 490 nm)/(mean control Abs 490 nM-mean blank Abs 490 nm) × 100.

### Plasma Protein Binding

Mouse plasma protein binding (PPB) was determined by equilibrium dialysis with 10 μM compound over 4 h at 37 °C. Compound recoveries were determined by HPLC-MS/MS.

### Animal Pharmacokinetic Study

Unrestrained, nonanesthetized mice (Female ICR (CD-1); Age 5–6 weeks; Size 22 ± 2 g; specific pathogen-free (SPF)) were injected IV with test article formulations via the tail vein. Animals were sacrificed by cardiac puncture under CO_2_ euthanasia at 0.08, 0.25, 1, 2, 4, 8, and 24 h post dosing. Blood was drawn into tubes coated with EDTA-K_3_, mixed gently and plasma was then harvested and kept frozen at −70 °C until further processing. To collect bronchoalveolar lining fluid (BALF), 0.5 mL of PBS was administered once through a tracheal cannula after which about 0.2 to 0.3 mL of BALF was obtained. The BALF was kept on ice and centrifuged within 1 h of collection and the supernatant was harvested and kept frozen at −70 °C. Plasma and BALF samples were analyzed by LC-MS/MS (AB SCIEX API 3000 mass spectrometer). The apparent ELF volume of each BALF sample was estimated using urea as an endogenous marker of the ELF dilution. The urea concentrations in BALF and plasma samples were determined with the QuantiChrom Urea Assay Kit (DIUR-100) (BioAssay Systems) following the manufacturer’s protocol. For each BALF sample, the drug concentration in ELF was calculated as follows: drug concentration_ELF_ = drug concentration_BALF_ × (urea concentration_plasma_/urea concentration_BALF_). The PK parameters of each compound after IV dosing (*t*_1/2_, C_0_, AUC_last_, and C_L_) were obtained from the noncompartmental analysis (NCA) of the plasma data using WinNonlin.

### Inhibition of LasB-Mediated Proteolysis of Immunoglobulin G (IgG)

This protocol was adapted from Cathcart et al.^25^ Briefly, 10 μg of IgG from human serum (Sigma) were incubated with 100 ng purified LasB in assay buffer (50 mM Tris-HCl pH 7.4, 2.5 mM CaCl_2_) for 4 h at 37 °C, with or without variable concentrations of inhibitor. Samples were then denatured and analyzed by 4–15% gradient SDS polyacrylamide gel electrophoresis (SDS-PAGE) and the gel stained using Coomassie blue. The IgG subunits are visualized as two bands at 28 kDa (light chain) and 54 kDa (heavy chain). Proteolysis by LasB results in the generation of a fragment of the heavy chain detected of approximately 37 kDa. Bands were quantified using ImageJ software, and IC_50_ values were determined using Prism software (GraphPad).

### Inhibition of LasB-Mediated Proteolysis of Pro-IL-1β-Derived Peptide

The peptide HDAPVRSLN, corresponding to amino acids 115–123 of the reference human pro-IL-1β sequence (UniProt: PO1584), was labeled on the N-terminus with Mca and on the C-terminus with Lys-Dnp (CPC Scientific) to generate an internally quenched fluorescent peptide (Sub115). Different concentrations of peptide were incubated in assay buffer (PBS, 0.01% Tween-20 or indicated buffers) and purified LasB with varying concentrations of inhibitor. The reaction was continuously monitored using a Victor multimode plate reader (PerkinElmer) with excitation at 355 nm and emission at 450 nm, and initial kinetic velocity was calculated. Inhibitory constants (*Ki*) were determined (two independent replicates) using a competitive inhibitor model.

### Inhibition of LasB Activation of IL-1β in *P. aeruginosa*-Infected THP-1 Macrophages

Human THP-1 macrophages were cultured by conventional methods then, pre-infection, culture media were replaced by RPMI lacking phenol red and antibiotics. Compounds VX-765 (caspase-1 inhibitor, developed by Vertex) and/or LasB inhibitors were added, immediately followed by inoculation with *P. aeruginosa* PAO1 or an isogenic *lasB* deletion mutant (Δ*lasB*), previously grown in Luria broth to late exponential phase (OD_600_ = 1.2) then washed and diluted in PBS, at a multiplicity of infection (MOI) of 10 to 1. After 2 h post-infection, 50 μL aliquots of supernatant were removed and used to quantitate both LasB activity and IL-1β functionality. To determine LasB activity, 50 μL of supernatant was added to Sub115 IL-1β peptide and hydrolysis of Sub115 was monitored spectrophotometrically with excitation at 355 nm and emission at 450 nm, as described above. Results were expressed as relative fluorescence units (RFU).

To determine IL-1β functionality, generation of an IL-1 signal was quantified by adding 50 μL of supernatant to HEK IL-1R-lux reporter cells cultured in RPMI supplemented with 5% heat-inactivated FBS, 200 μg/mL penicillin/streptomycin, 200 μg/mL hygromycin, 100 μg/mL zeomycin, and 0.5 μg/mL puromycin. No bacteria survived in this antibiotic condition; 18 h post-treatment, IL-1β-dependent luciferase induction was assayed by luminescence measured with d-luciferin substrate (Steady-Luc, Biotium) on a multimode reader (PerkinElmer) and expressed as relative luminescence units (RU), as previously described.^16^

In the first variation, experimental infections were performed with *P. aeruginosa* PAO1 and Δ*lasB* bacteria incubated with varying quantities of inhibitor at final concentrations ranging from 0.5 to 8 μM. In the second, cells were treated and infected in the same manner and also treated with 5 μM VX-765 to provide a control for IL-1β activation by the inflammasome pathway.

### Mouse *P. aeruginosa* Lung Infection Model

Infection experiments were approved by the Institutional Animal Care and Use Committee of Emory University. To prepare the bacterial inoculum, *P. aeruginosa* PAO1 (or its Δ*lasB* derivative) was grown in Luria broth with shaking at physiologic (37 °C) temperature for 18 h to maximally express LasB (at late log phase). Bacteria were then harvested, washed, and diluted in sterile phosphate-buffered saline (PBS) for delivery of 10^9^ colony-forming units (CFUs) in 40 μL. For infection, female C57Bl/6 mice (five/group) were sedated using isofluorane and the bacterial suspension was slowly administered via micropipette between the nostrils and the animals were allowed to aspirate the inoculum via the normal breathing process. Compounds **12** and **16** were administered in PBS via injection (100 μL volume) to achieve doses of 10 and 30 mg/kg. The mice were euthanized 24 h post-infection and the lungs were excised. Active IL-1β was quantified by adding 100 μL of cell-free homogenate to IL-1β responsive HEK IL-1R-lux reporter cells. Activity was assayed by luminescence measured with d-luciferin substrate (Steady-Luc, Biotium) on a multimode reader (PerkinElmer) and expressed as relative luminescence units (RU). CFU was enumerated by dilution plating on Luria agar. Statistics were calculated by ANOVA with a Dunnett post-test.

## Supporting Information Available

Dixon plots of compounds **12** and **16** (Figure S1); Western blots showing quantification of LasB variants (Figure S2); and synthetic experimental and X-ray data (PDF)

## Accession Codes

The structure of compound **16** in the LasB activity has been assigned PDB code 7QH1. The authors will release the atomic coordinates and experimental data upon article publication.
