# Supplementary material for: Chemical Optimization of Selective Pseudomonas aeruginosa LasB Elastase Inhibitors and Their Impact on LasB-Mediated Activation of IL-1β in Cellular and Animal Infection Models
Source: ACS Infect Dis. 2023 Jan 20;9(2):270–82. doi: 10.1021/acsinfecdis.2c00418 (PMC9926489; doi:10.1021/acsinfecdis.2c00418)
Supplement: Supplementary file 1 — id2c00418_si_001.pdf [file id2c00418_si_001.pdf]

Supporting Information

Chemical optimization of selective *Pseudomonas aeruginosa* LasB elastase inhibitors and their impact on LasB-mediated activation of IL-1β in cellular and animal infection models

Martin J. Everett[1]*, David T. Davies[1], Simon Leiris[1], Nicolas Sprynski[1], Agustina Llanos[1], Jérôme M. Castandet[1], Clarisse Lozano[1], Christopher N. LaRock[2], Doris L. LaRock[2], Giuseppina Corsica[3], Jean-Denis Docquier[3,4], Thomas D. Pallin[5], Andrew Cridland[5], Toby Blench[5], Magdalena Zalacain[1] and Marc Lemonnier[1]

[1] Antabio SAS, Biostep, 436 rue Pierre et Marie Curie, 31670 Labège, France

[2] Department of Microbiology and Immunology, Rollins Research Center, 1510 Clifton Rd, Atlanta, Georgia 30322, USA

[3] Dipartimento di Biotecnologie Mediche, Università degli Studi di Siena, Viale Bracci 16, 53100 Siena, Italy

[4] Centre d'Ingénierie des Protéines - InBioS; University of Liège, Allée du six Août 11, 4000 Liège, Belgium

[5] Charles River Laboratories, 8-9 The Spire Green Centre, Harlow, Essex, CM19 5TR, UK

Pages: 44

Figures: 7

Contents

Supplementary figures……Pages SS3

Synthetic experimental……Pages SS-42

Protein crystallography……Pages S43-S44

* Author for correspondence, martin.everett@antabio.com

Supplementary Figures

A

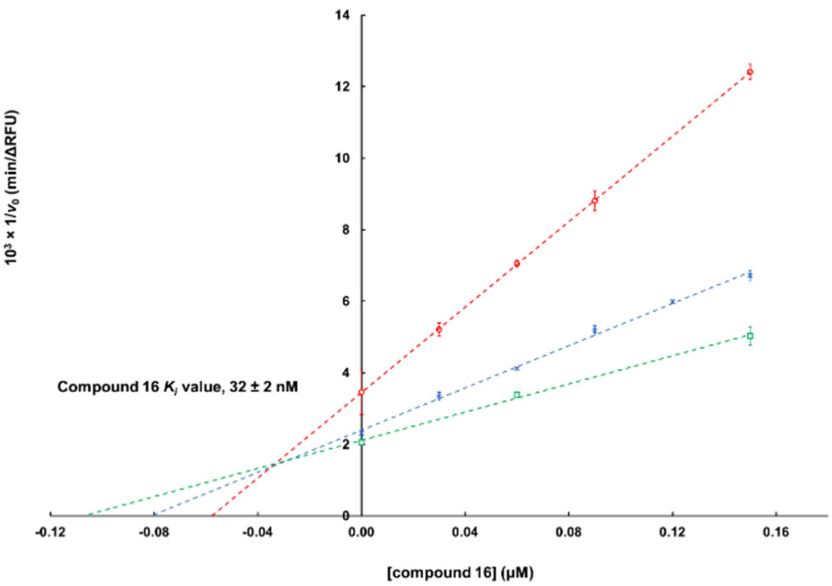

B

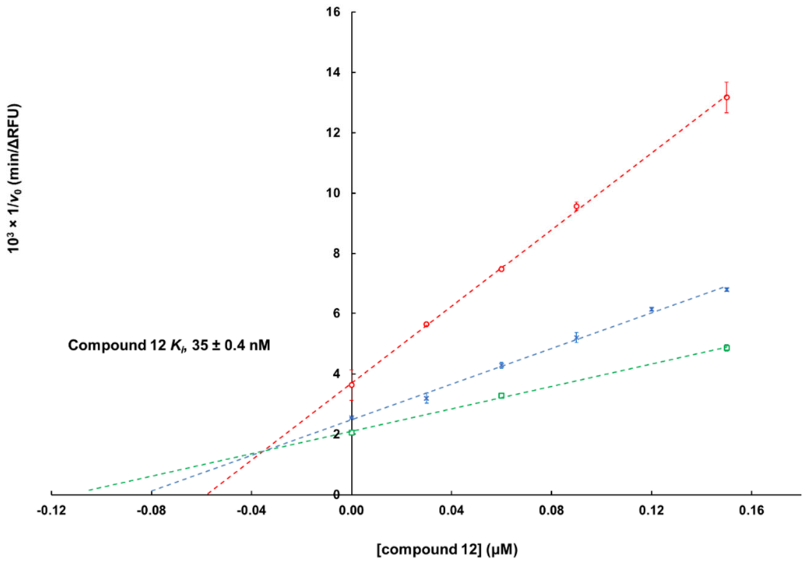

Fig. S1. Compounds 12 and 16 are competitive inhibitors of LasB. Dixon plots of compounds **12** (A) and **16** (B). X-axis displays the concentration of inhibitor and y-axis displays the reciprocal of the initial velocity (reaction rate, ΔRFU/min). The different substrate concentrations are shown by the coloured points/line; 100 μM (red), 250 μM (blue), 1500 μM (green). The point of intersection = **-** K_i (μM); K_i values determined as 35 nM for compound **12** and 32 nM for compound **16**.

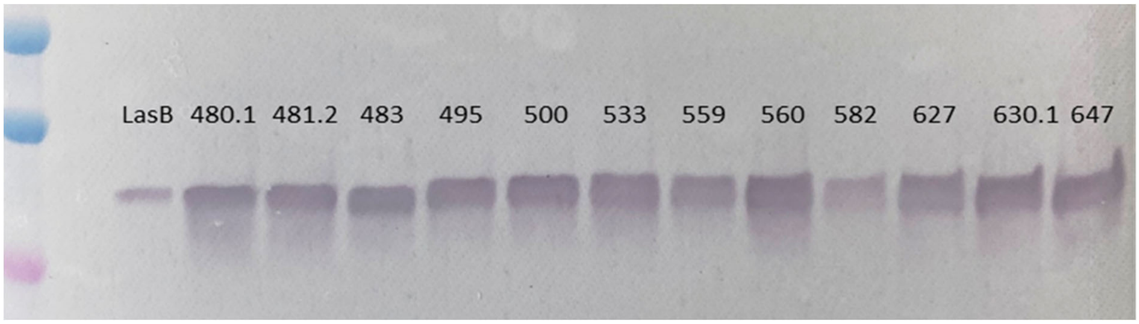

Strain	Ctrl[1]	480.1	481.2	483	495	500	533	559	560	582	627	630.1	647
Variant	WT	WT	WT	5 aa[2]	S241G	S241G	T651I	Q71L	S460T	5 aa[2]	Q71L	S241G	5 aa[2]
Band density	7993	38203	30096	28568	27475	31455	31103	20837	32379	12438	26637	37311	37589
Amount (ng)	5	23.9	18.8	17.9	17.2	19.7	19.5	13.0	20.3	7.8	16.7	23.3	23.5
Conc. (ng/µl)	0.5	2.39	1.88	1.79	1.72	1.97	1.95	1.30	2.03	0.78	1.67	2.33	2.35

[1] LasB protein control (5 ng). [2] 5 aa: five amino acid changes; Q102R, S241G, D244N, K282N, R471S.

Fig. S2. **LasB variants produced by clinical *P. aeruginosa* strains show similar expression levels in culture supernatants, as determined by SDS-PAGE Western blot probed with LasB polyclonal antibody.** The amount of LasB protein in 10 µl of culture supernatant was estimated by comparing band density to that of 5ng purified LasB.

Experimental for the Synthesis of Compounds 1-16

General:1H NMR spectra are reported at 300, 400 or 500 MHz in DMSO-d6 solutions (δ in ppm), using DMSO-d_5 as reference standard (2.50 ppm), or $CDCl_3$ solutions using chloroform as the reference standard (7.26 ppm). When peak multiplicities are reported, the following abbreviations are used: s (singlet), d (doublet), t (triplet), m (multiplet), bs (broadened singlet), bd (broadened doublet), dd (doublet of doublets), dt (doublet of triplets), q (quartet). Coupling constants, when given, are reported in hertz (Hz). The term "purified by prep hplc (MDAP)" refers compound purification using a mass-directed auto purification system on an Agilent 1260 infinity machine with an XSelect CHS Prep C18 column, eluting with 0.1% FA in water/ACN and detection with a Quadrupole LC/MS.

Abbreviations: ACN – acetonitrile; aq. – aqueous; Bpin – Bis(pinacolato)diboron; CaCl2 – calcium chloride; Cu(OAc)2 – copper(II) acetate; DCM – dichloromethane; DIPEA – N,N-diisopropylethylamine; DMF – N,N-dimethylformamide; dppf - 1,1'-bis(diphenylphosphino)ferrocene; EDC.HCl – N-(3-dimethylaminopropyl)-N'-ethylcarbodiimide hydrochloride; Et2O – diethyl ether ; EtOAc – ethyl acetate; EtOH – ethanol ; Et3N – triethylamine ; FA – formic acid ; h – hour(s) ; HATU - 1-[bis(dimethylamino)methylene]-1H-1,2,3-triazolo[4,5-b]pyridinium 3-oxid hexafluorophosphate; HCl -hydrochloric acid; HOBT – hydroxybenzotriazole; H2SO4 – sulfuric acid; K3PO4 – potassium phosphate; MeOH – methanol; min – minute(s); MgSO4 – magnesium sulfate; MTBE – methyl *tert*-butyl ether; NBS – N-bromosuccinimide; NaHCO3 – sodium bicarbonate; NaHDMS – sodium bis(trimethylsilyl)amide; Na2SO4 – sodium sulfate; NMM – N-methylmorpholine; (dba)3 - tris(dibenzylideneacetone)dipalladium(0); PdCl2(dppf) - [1,1′-bis(diphenylphosphino)ferrocene]dichloropalladium(II); RT – room temperature; Ruphos Pd G1 - chloro-(2-dicyclohexylphosphino-2′,6′-diisopropoxy-1,1′-biphenyl)[2-(2-aminoethyl)phenyl]palladium(II); SCX-2 - Strong cation exchange resin (silica-propyl sulfonic acid); TES – triethylsilane; TFA – trifluoroacetic acid; THF – tetrahydrofuran; T3P - propylphosphinic anhydride

Compound 16:

2-(2-(((5-(3-(bis(2-hydroxyethyl)(methyl)ammonio)propoxy)-6-methoxybenzo[d]thiazol-2-yl)methyl)carbamoyl)-5,6-difluoro-2,3-dihydro-1H-inden-2-yl)acetate

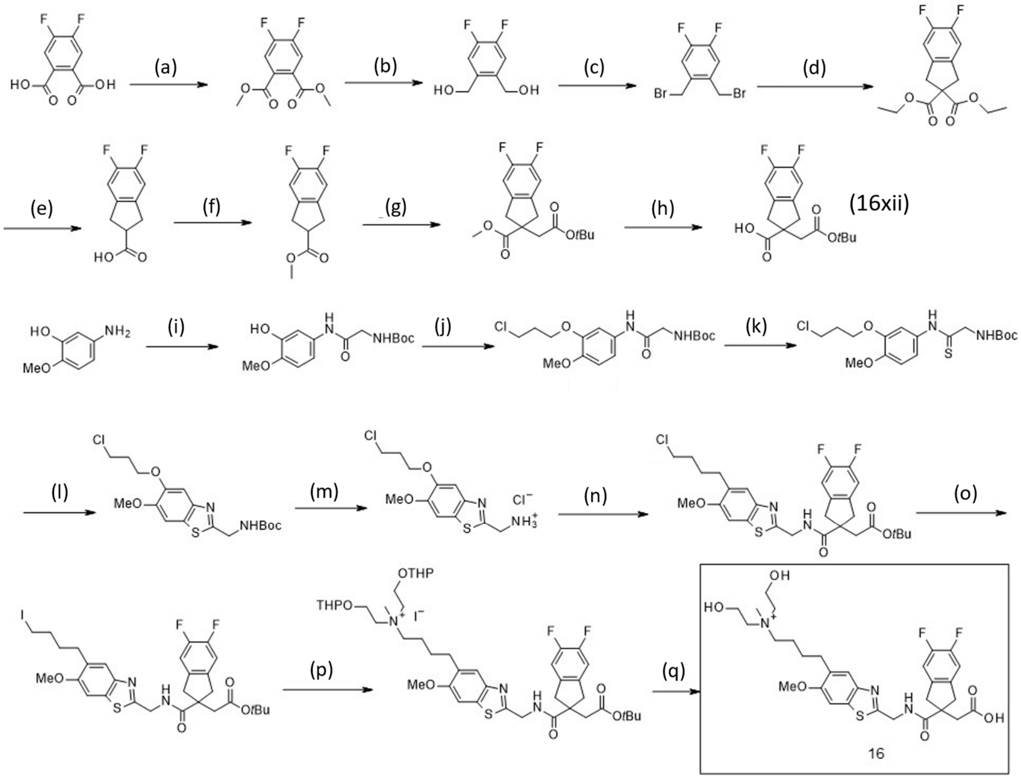

Fig. S3; Synthesis of Compound 16

a. H2SO4, MeOH b. LiAlH4 c. HBr d. NaH, diethylmalonate e. HCl f. H2SO4, MeOH g. NaHMDS, BrCH2CO2t-Bu h. LiOH i. CDI, BOCNHCH2CO2H j. Cl(CH2)3Br, K2CO3 k. P4S10, Na2CO3 l. K3Fe(CN)6, NaOH m. HCl n. EDC.HCl, HOBT, NMM, (16xii) o. KI p. (THPOCH2CH2)2NMe q. TFA

i. *tert*-butyl (2-((3-hydroxy-4-methoxyphenyl)amino)-2-oxoethyl)carbamate

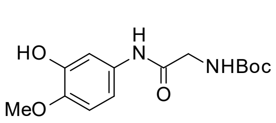

1,1´-Carbonyldiimidazole (51.73 g, 319 mmol) was added portion wise to a suspension of *N*-BOC-glycine (55.90 g, 319.00 mmol) in dry THF (560 mL) at 20°C and the mixture was stirred for 2h. The resulting solution was added dropwise over a period of 3h to a stirred solution of 5-amino-2-methoxyphenol (44.40 g, 319.00 mmol) at 0 °C in dry THF (1.3 L) then stirred for 18h at RT. The THF was removed in vacuo and the residue partitioned between water (1 L) and EtOAc (1 L). The phases were separated and the organic phase was washed sequentially with 1M aqueous hydrochloric acid solution (1 L), saturated aqueous sodium bicarbonate solution (1 L) then saturated brine solution (500 mL). The original aqueous phase was extracted with MTBE (500 mL) and the organic extract was further used to extract the acidic and basic washes, then washed with saturated brine solution (250 mL). The combined EtOAc and MTBE extracts were dried over sodium sulfate, filtered and evaporated *in vacuo* to give a dark red foam. This was dissolved in EtOAc (180 mL) and heptane (180 mL) was slowly added. This was stirred for 72h, diluted with more heptane (360 mL) and stirred for a further 4h. Filtration and drying afforded a light brown powder (76.g, 81%).

^{1}H NMR (400 MHz, $CDCl_3$) δ 8.16 (brs, 1H), 7.09 (m, 1H), 6.97 (m, 1H), 6.75 (m, 1H), 5.94 (s, 1H), 5.34 (br s, 1H), 3.89 (s, 2H), 3.84 (s, 3H), 1.45 (s, 9H).

LCMS M/z 297.0 $[M+H]^+$.

ii. *tert*-butyl (2-((3-(3-chloropropoxy)-4-methoxyphenyl)amino)-2-oxoethyl)carbamate

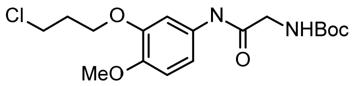

Potassium carbonate (22.60 g, 163.80 mmol) and then 1-bromo-3-chloropropane (16.2 mL, 163.60 mmol) to a solution of *tert*-butyl (2-((3-hydroxy-4-methoxyphenyl)amino)-2-oxoethyl)carbamate (32.30 g, 109.10 mmol) in EtOH (325 mL). The mixture was heated to reflux for 18h then allowed to cool to ambient temperature the EtOH was removed *in vacuo*. The residue was partitioned between water and MTBE (400 mL/400 mL). The phases were separated and the aqueous phase further extracted with MTBE. The combined organic extracts were washed with saturated brine solution, dried over Na_2SO_4 and evaporated *in vacuo*. The residue was stirred with heptane for 72h then the resulting solid collected by filtration and dried *in vacuo* at 45°C affording a pale pink solid (37.0g, 91%). Filtration and drying afforded a beige coloured powder (29.3g, 72%).

^{1}H NMR (400 MHz, $CDCl_3$) δ 8.13 (brs, 1H), 7.28 (m, 1H), 6.88 (m, 1H), 6.78 (m, 1H), 5.30 (brs, 1H), 4.12 (t, *J* = Hz, 2H), 3.92 (s, 2H), 3.81 (s, 3H), 3.74 (t, *J* = 6.36 Hz, 2H), 2.26 (m, 2H), 1.44 (s, 9H).

LCMS M/z 373.0 $[M+H]^+$.

iii. *tert*-Butyl (2-((3-(3-chloropropoxy)-4-methoxyphenyl)amino)-2-thioxoethyl)carbamate

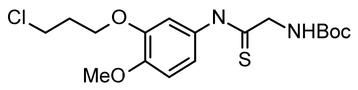

Sodium carbonate (2.6 g, 24.5 mmol) was added in one portion to a solution of phosphorus pentasulphide (P4S10), (10.9 g, 24.4 mmol), in THF (375 mL). The mixture was stirred for 1h, until complete solution was achieved. *tert*-Butyl (2-((3-(3-chloropropoxy)-4-methoxyphenyl)amino)-2-oxoethyl)carbamate (36.4 g, 97.7 mmol) was added in one portion and the mixture heated at 65°C and for 3.5h, then allowed to cool to ambient temperature the THF was removed *in vacuo*. DCM (500 mL) was added and the suspension filtered through celite, rinsing through with DCM. The collected solid was further stirred with DCM and the suspension filtered through celite, rinsing through with DCM. The combined filtrates were evaporated to give a dark oil (44g) which was suspended in MTBE (220 mL) and stirred for 1h at 25°C, then the resultant solid was removed by filtration, washing the residue with MTBE (2x25 mL). The combined MTBE filtrates were evaporated *in vacuo* and the residue treated with heptane (200 mL) and stirred for 18h. The resulting solid was filtered and dried *in vacuo* affording a beige coloured powder (29.3 g, 72%).

^{1}H NMR (400 MHz, $CDCl_3$) δ 7.61 (d, *J* = 2.48 Hz, 1H), 7.11 (m, 1H), 6.84 (m, 1H), 4.27 (s, 2H), 4.14 (t, *J* = 5.92 Hz, 2H), 3.83 (s, 3H), 3.74 (t, *J* = 6.32 Hz, 2H), 2.26 (m, 2H), 1.46 (s, 9H).

LCMS M/z 388.9 $[M+H]^+$.

iv. *tert*-butyl ((5-(3-chloropropoxy)-6-methoxybenzo[d]thiazol-2-yl)methyl)carbamate

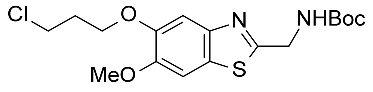

Potassium ferricyanide (94.00 g, 286.00 mmol) and sodium hydroxide (22.9 g, 571 mmol) were suspended in water (550 mL). The mixture was heated to 35°C and stirred for 1h. A solution of *tert*-butyl (2-((3-(3-chloropropoxy)-4-methoxyphenyl)amino)-2-thioxoethyl)carbamate (27.60 g, 71.00 mmol) in MeOH (275 mL) was added dropwise over 2h. The resulting mixture was stirred at 35°C for a further 2h and concentrated under reduced pressure. EtOAc (300 mL) was added to the residue and the mixture washed with water (250 mL). The aqueous phase was extracted with EtOAc (300 mL). The organic extracts were combined, washed with saturated brine solution (100 mL), dried over sodium sulfate, filtered and concentrated under reduced pressure to give an orange solid (26.50 g). The solid was recrystallised from IPA (185 mL) affording an orange solid (19.5 g, 71%).

^{1}H NMR (400 MHz, $CDCl_3$) δ 7.45 (s, 1H), 7.24 (s, 1H), 5.38 (br s, 1H), 4.68 (d, *J* = 6.0 Hz, 2H), 4.21 (t, *J* = 6.0 Hz, 2H), 3.90 (s, 3H), 3.77 (t, *J* = 6.36 Hz, 2H), 2.32 (m, 2H), 1.49 (s, 9H).

LCMS M/z 387.0 $[M+H]^+$.

v. 2-aminomethyl-5-(3-chloropropoxy)-6-methoxybenzo[d]thiazole hydrochloride

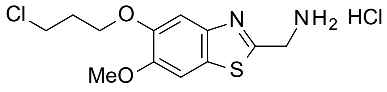

Tert-butyl ((5-(3-chloropropoxy)-6-methoxybenzo[d]thiazol-2-yl)methyl)carbamate (3.86 g, 10.00 mmol) was suspended in 5M hydrogen chloride in IPA (40 mL). The reaction mixture was heated to 50°C for 0.75h giving a thick suspension, which was diluted with EtOAc (50 mL). The solid was filtered, washed with EtOAc (25 mL) and dried *in vacuo* at 45°C for 18h affording a beige coloured powder (3.40 g, 100%).

^{1}H NMR (400 MHz, DMSO D_6) δ 8.80 (br s, 3H), 7.73 (s, 1H), 7.53 (s, 1H), 4.49 (m, 2H), 4.17 (m, 2H), 3.84 (s, 3H), 3.83 (m, 2H), 2.19 (m, 2H).

LCMS M/z 287.0 $[M+H]^+$.

vi. [4,5-difluoro-2-(hydroxymethyl)phenyl]methanol

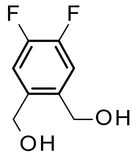

BH_3.THF complex 1.0M in THF (1.48 L, 1.48 mol) was added to a stirred suspension of 4, 5-difluorophthalic acid (100 g, 0.495 mol) in THF (500 mL) over a period of 2h at -10°C under argon. The reaction mixture was stirred at RT for 4h, cooled to -10°C and quenched by slow addition of methanol/acetic acid (9:1, 1 L) over 1h. The reaction mixture was concentrated under reduced pressure and resulting residue was diluted with EtOAc (1 L), washed with sat. $NaHCO_3$ and brine. The organic layer was concentrated under reduced pressure and the residue was triturated with n-pentane (2 x 400 mL) to give a white solid (70 g, 82%).

^{1}H NMR (400 MHz, d6-DMSO) δ 7.35 (t, *J* = 10 Hz, 2H), 5.27 (t, *J* = 5.2 Hz, 2H), 4.47 (d, *J* = 5.6 Hz, 4H).

LCMS M/z 175.1 $[M+H]^+$.

vii. 1,2-bis(bromomethyl)-4,5-difluoro-benzene

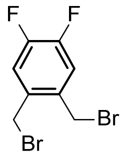

A mixture of [4,5-difluoro-2-(hydroxymethyl)phenyl]methanol (20 g, 0.115 mol) and 48% hydrobromic acid (100 mL) was heated to 110°C for 10h. The reaction mixture was cooled to RT and water (100 mL) was added followed by DCM (200 mL). The organic layer and washed with saturated aqueous $NaHCO_3$, water and brine. The organic layer was dried with sodium sulfate and evaporated under reduced pressure at below 40°C affording a brown oil (30 g, 87%). The crude compound was used as such in the next step without further purification.

^{1}H NMR (400 MHz, DMSO D_6) δ 7.61-7.64 (m, 2H), 4.78 (s, 4H).

LCMS M/z 301.1 $[M+H]^+$.

viii. diethyl 5,6-difluoroindane-2,2-dicarboxylate

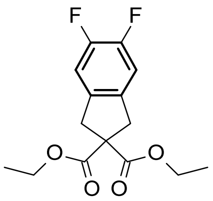

A solution of diethylmalonate (26.7g, 167 mmol) in THF (120 mL) was added to a stirred suspension of NaH (14.6 g, 60% dispersion in oil by weight, 365 mmol of NaH) in dry THF (265 mL) over 2.5 h at -10°C under argon. The reaction mixture was stirred for 0.5h then a solution of 1,2-bis(bromomethyl)-4,5-difluoro-benzene (50g, 167 mmol) in THF (120 mL) was added over 2h at -10°C. The reaction mixture was stirred at RT for 4h and quenched by adding 1N HCl (120 mL) at -10°C for 1h. The organic layer was separated, and the aqueous layer was extracted with EtOAc (2 x 60 mL). The combined organic layer was washed with water and concentrated under reduced pressure affording a pale brown liquid (50g, approx.100%). The crude compound was used as such in the next step without purification.

^{1}H NMR (500 MHz, $CDCl_3$) δ 6.97 (t, *J* = 9 Hz, 2H), 4.21 (q, *J* = 14 Hz and 7 Hz 4H), 3.52 (s, 4H), 1.23 (t, *J* = Hz).

LCMS M/z 299.2 $[M+H]^+$.

ix. 5,6-difluoroindane-2-carboxylic acid

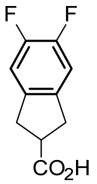

Water (225 mL) and concentrated HCl (225 mL) were added to a solution of diethyl 5,6-difluoroindane-2,2-dicarboxylate (45 g, 0.15 mol) in dioxane (225 mL) at RT. The reaction mixture was heated at 110°C for 30h, then cooled to RT and water (225 mL) was added. The resulting solid was isolated by filtration. The residue was triturated with 10% EtOAc in hexane (450 mL) affording an off-white solid (19.5 g, 65%).

^{1}H NMR (400 MHz, DMSO D_6) δ 12.35 (s, 1H), 7.27 (t, *J* = 9.2 Hz, 2H), 3.36-3.27 (m, 1H), 3.15-3.04 (m, 4H).

LCMS M/z 197.0 $[M-H]^-$.

x. **methyl 5,6-difluoroindane-2-carboxylate**

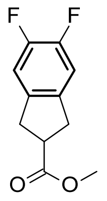

Concentrated H_2SO_4 (1.5 mL) was added to a stirred solution of 5,6-difluoroindane-2-carboxylic acid (15 g, 0.076 mol) in MeOH (150 mL) over 15 minutes at 0° C. The reaction mixture was heated at 65°C for 2h then evaporated under reduced pressure. The resulting residue was co-distilled with MTBE (30 mL) and diluted with MTBE (150 mL). The acidity was adjusted to pH 7 – 8 using 1N aq. NaOH solution. The organic layer was washed with water (45 mL) followed by brine (45 mL), dried with sodium sulfate, filtered and concentrated under reduced pressure at below 50°C affording a light brown solid (14.5 g, 91%).

^{1}H NMR (400 MHz, $CDCl_3$) δ 7.03-6.95 (m, 2H), 3.72 (s, 3H), 3.42-3.33 (m, 1H), 3.24-3.11 (m, 4H).

LCMS M/z 213.1 $[M+H]^+$.

xi. **methyl 2-(2-*tert*-butoxy-2-oxo-ethyl)-5,6-difluoro-indane-2-carboxylate**

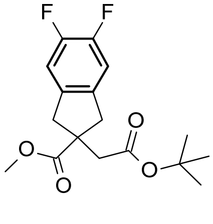

A solution of sodium bis(trimethylsilyl)amide (305 mL, 2M in THF, 0.61 mol) was added to a stirred solution of methyl 5,6-difluoroindane-2-carboxylate (100 g, 0.471 mol) in THF (1 L) over 0.75h at -70°C under argon. The reaction mixture was stirred for 1h. Then a solution of *tert*-butyl bromoacetate (101g, 0.52 mol) in THF (100 mL) was added over 1h at -70°C and stirred for 1h. Brine (1 L) was added at -70ºC and the resulting mixture was stirred at RT for 1h. The aqueous layer was extracted with EtOAc (2 x 500 mL) and combined organic extracts were washed with brine (500 mL), dried with sodium sulfate, filtered and concentrated under reduced pressure. The residue was co-distilled with hexane (200 mL) and

stirred in hexane (100 mL) for 1 – 2h at 0 – 10°C. The resulting solid was filtered, washed with hexane (50 mL) and dried *in vacuo* to afford a light pink solid (70 g, 46%).

^{1}H NMR (400 MHz, $CDCl_3$) δ 6.98-6.94 (m, 2H), 3.72 (s, 3H), 3.49-3.45 (m, 2H), 2.92-2.88 (m, 2H), 2.71 (s, 2H), 1.49 (s, 9H).

LCMS M/z 327.2 $[M+H]^+$.

xii. 2-(2-*tert*-butoxy-2-oxo-ethyl)-5,6-difluoro-indane-2-carboxylic acid

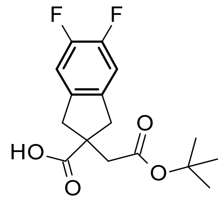

$LiOH.H_2O$ solid (97g, 2.3 mol) was slowly added to a stirred solution of methyl 2-(2-*tert*-butoxy-2-oxo-ethyl)-5,6-difluoro-indane-2-carboxylate (600 g, 1.92 mol) in THF (1.5 L), ethanol (1.5 L) and water (3 L) at RT. The reaction mixture was stirred for 2h, concentrated under reduced pressure and the resulting aqueous solution was washed with MTBE (2 x 3 L). The aqueous layer was acidified with 1N HCl to pH 4 – 5 at 10 – 20° C, stirred for 15 minutes and extracted with MTBE (2 x 3 L). The combined organic extracts were dried over sodium sulfate and evaporated under reduced pressure. The resulting crude material was stirred in 20% MTBE in hexane (3 L) for 2h at 30°C, filtered, washed with 20% MTBE in hexane (600 mL) and dried *in vacuo* at 50°C to afford a brown solid (430 g, 75%).

^{1}H NMR (400 MHz, DMSO D_6) δ 12.4 (brs, 1H), 7.26 (t, J = 9.2 Hz, 2H), 3.52-3.31 (m, 2H), 2.92-2.88 (m, 2H), 2.70 (s, 2H), 1.37 (s, 9H).

LCMS M/z 311.1 $[M-H]^-$.

xiii. *tert*-butyl 2-[2-[[5-(3-chloropropoxy)-6-methoxy-1,3-benzothiazol-2-yl]methylcarbamoyl]-5,6-difluoro-indan-2-yl]acetate

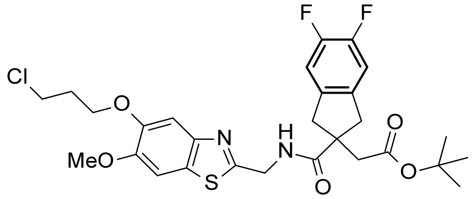

A solution of 2-(2-*tert*-butoxy-2-oxo-ethyl)-5,6-difluoro-indane-2-carboxylic acid (495 g, 1.58 mol), EDC.HCl (455.4 g, 2.38 mol), HOBT.H_2O (242.6 g, 1.58 mol) and NMM (871 mL) in ACN (5 L) was treated with 2-aminomethyl-5-(3-chloropropoxy)-6-methoxybenzo[d]thiazole hydrochloride (590 g, 1.64 mol) portion wise over 0.75h. The reaction mixture was stirred at RT for ~20 h. Water (5 L) was added portion wise over 0.75h and the resulting slurry was stirred at RT for 1h. The mixture was filtered and the residue was washed with water (2 x 5 L) and dried *in vacuo* at 45°C, giving a brown solid (826 g, 90%).
^{1}H NMR (400 MHz, $CDCl_3$) δ 7.74 (s, 2H), 7.43 (s, 1H), 7.27-7.23 (m, 2H), 7.04-6.99 (m, 3H), 4.78 (d, *J* = 5.6 Hz, 2H), 4.24 (t, *J* = 6 Hz, 2H), 3.92 (s, 3H), 3.81 (t, *J* = 6 Hz, 2H), 3.48 (d, *J* = 16.4 Hz, 2H), 3.08 (d, *J* = 16.4 Hz, 2H), 2.77 (s, 2H), 2.38-2.31 (m, 2H), 1.40 (s, 9H).
LCMS M/z 581.1, 583.1 $[M+H]^+$.

xiv. *tert*-butyl 2-[2-[[5-(3-iodopropoxy)-6-methoxy-1,3-benzothiazol-2-yl]methylcarbamoyl]-5,6-difluoro-indan-2-yl]acetate

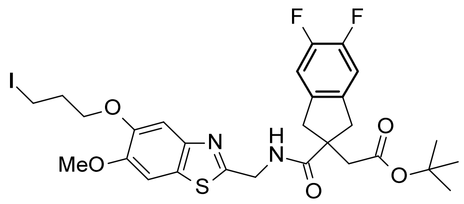

A mixture of *tert*-butyl 2-[2-[[5-(3-chloropropoxy)-6-methoxy-1,3-benzothiazol-2-yl]methylcarbamoyl]-5,6-difluoro-indan-2-yl]acetate (398 g, 0.68 mol) and NaI (513.4 g, 3.42 mol) in 2-butanone (3.98 L) was heated to 70-80° C for 8h. The reaction was cooled to RT then water (2 L) was added portion wise and the mixture stirred for 1h until complete dissolution occurred. The phases were separated, and the organic extract was washed with brine (2L) then dried over $MgSO_4$, filtered and concentrated under reduced pressure. Heptane (3 x 400 mL) was added to the residue and the mixture was re-concentrated under reduced pressure to give a pale orange solid, which was chromatographed on silica eluting with 0-15% EtOAc in DCM to give a beige solid (347.3 g, 74%).
^{1}H NMR (400 MHz, $CDCl_3$) δ 7.43 (s, 1H), 7.27 (s, 1H), 7.23 (d, *J* = 3.6 Hz, 1H), 7.05-6.99 (m, 3H), 4.78 (d, *J* = 5.6 Hz, 2H), 4.16 (t, *J* = 6 Hz, 2H), 3.92 (s, 3H), 3.50-3.41 (m, 4H), 3.06 (d, *J* = 16.4 Hz, 2H), 2.77 (s, 2H), 2.41-2.35 (m, 2H), 1.40 (s, 9H).
LCMS M/z 673.0 $(M+H)^+$.

xv. N-methyl-2-tetrahydropyran-2-yloxy-N-(2-tetrahydropyran-2-yloxyethyl)ethanamine

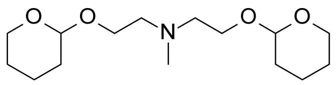

A mixture of p-TSA monohydrate (2.1 kg, 11.0 mol) and toluene (11.9 L) was treated with *N*-methyldiethanolamine (1.14 L, 9.93 mol) over ~0.5h while maintaining the internal temperature <30°C with external cooling. The reaction mixture was then heated at reflux with a Dean-Stark apparatus until no further water was removed. The reaction was then cooled to 10 C and 3,4-dihydro-2H-pyran (3.0 L, 32.8 mol) was added dropwise over 3 h while maintaining the internal temperature <40°C. The reaction was then stirred at 20°C for 2.5 h. K_2CO_3 (2.1 kg, 15.1 mol) was then added portion wise. This was followed by the addition of a solution of $NaHCO_3$ (750 g) in water (8 L) which resulted in extensive foaming. Water (8 L) was then added and stirred at 15-25°C until complete dissolution had occurred. The phases were then separated, and the organic phase was dried with $MgSO_4$, filtered and concentrated under reduced pressure. This gave a yellow oil (3.6 kg) which was purified using a silica pad (20 kg) eluting with 1:1 EtOAc:heptane then 1:1 EtOAc:heptane +5% triethylamine. The product-containing fractions were concentrated under reduced pressure to give a pale-yellow oil (1.97 kg, 71%).

^{1}H NMR (400 MHz, $CDCl_3$) δ 4.60 (t, J = 3.2 Hz, 2H), 3.87-3.84 (m, 4H), 3.54-3.51 (m, 4H), 2.71-2.69 (m, 4H), 2.36 (s, 3H), 1.86-1.70 (m, 2H), 1.69-1.61 (m, 2H), 1.54-1.51 (m, 8H). LCMS M/z 288.1 $(M+H)^+$.

xvi. 3-[[2-[[[2-(2-*tert*-butoxy-2-oxo-ethyl)-5,6-difluoro-indane-2-carbonyl]amino]methyl]-6-methoxy-1,3-benzothiazol-5-yl]oxy]propyl-methyl-bis(2-tetrahydropyran-2-yloxyethyl)ammonium iodide

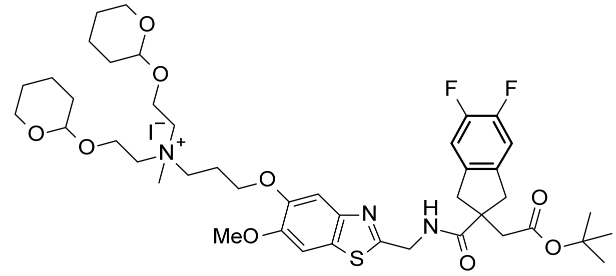

A suspension of *tert*-butyl 2-[2-[[5-(3-iodopropoxy)-6-methoxy-1,3-benzothiazol-2-yl]methylcarbamoyl]-5,6-difluoro-indan-2-yl]acetate (460 g, 0.68 mol) in ACN (4.6 L) was treated portion wise with N-methyl-2-tetrahydropyran-2-yloxy-N-(2-tetrahydropyran-2-yloxyethyl)ethanamine (275.2 g, 0.96 mol). The mixture was heated to 70°C for 21h (solution occuring at 40 °C). The reaction mixture was cooled and concentrated under reduced pressure to give a dark orange oil. DCM was then added and the mixture re-concentrated under reduced pressure to give a pale orange foam (0.8 kg) which was used without purification.
^{1}H NMR (400 MHz, d6-DMSO) δ 8.65 (bs, 1H), 7.58 (s, 1H), 7.48 (s, 1H), 7.26 (m, 2H), 4.66 (m, 2H), 4.58 (m, 2H), 4.11-3.25 (m, 18H), 3.15 (s, 3H), 2.99 (m, 2H), 2.71 (s, 2H), 2.25 (m, 2H), 1.63 (m, 4H), 1.47 (m, 6H), 1.31 (s, 9H).
LCMS M/z 832 (M^+).

xvii. 2-[2-[[5-[3-[bis(2-hydroxyethyl)-methyl-ammonio]propoxy]-6-methyl-1,3-benzothiazol-2-yl]methylcarbamoyl]-5,6-difluoro-indan-2-yl]acetate

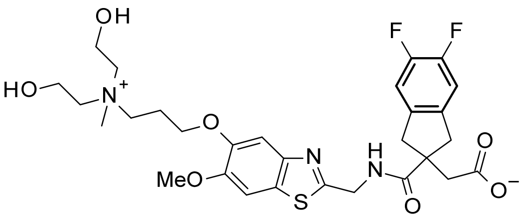

A solution of crude 3-[[2-[[[2-(2-*tert*-butoxy-2-oxo-ethyl)-5,6-difluoro-indane-2-carbonyl]amino]methyl]-6-methoxy-1,3-benzothiazol-5-yl]oxy]propyl-methyl-bis(2-tetrahydropyran-2-yloxyethyl)ammonium iodide (296 g) in DCM (591 mL) was cooled to 10-15°C then TFA (887 mL) was then added dropwise over ~10 minutes, (note - there was an exotherm over first half of addition). The reaction was then stirred at RT for 2.5h then added dropwise to TBME (17.7 L) which resulted in the precipitation of a yellow solid. The mixture

was evaporated under reduced pressure then more TBME (5.91 L) was added to the solid. The suspension was stirred for 5 minutes allowed to settle and the supernatant decanted off. This was repeated twice with TBME (2 x 3.25 L) then TBME (3.25 L) was added and the suspension was filtered under nitrogen. The solid was washed with TBME (1.92 L) then dried under vacuum at 20°C overnight, affording the TFA salt of the title product as a dark orange solid (258.6 g). This TFA salt was split into 3 portions; 100 g (94.1 g free base equivalent), 100 g (94.1 free base equivalent) and 58.6 g (55.2 g free base equivalent) which were charged to 3 separate flasks. EtOH (500 mL, 500 ml and 250 mL respectively) was added followed portion wise by DIPEA (0.38 mL/g of TFA salt) which adjusted the acidity to pH8. The solutions were stirred at RT. After 5h, the suspensions were combined, filtered and the filter cake was washed with EtOH/DIPEA (487 mL/24.3 mL) then EtOH (243 mL). The filter cake was dried, affording the title zwitterionic product as a pale-yellow solid (118.7 g, 64%).
^{1}H NMR (400 MHz, d6-DMSO) δ 12.8 (s, 1H), 7.62 (s, 1H), 7.51 (s, 1H), 7.21 (t, J = 7.2 Hz, 2H), 5.45 (s, 2H), 4.60 (d, J = 4.4 Hz, 2H), 4.10 (t, J = 4.8 Hz, 2H), 3.86-3.82 (m, 4H), 3.78 (s, 3H), 3.60-3.54 (m, 2H), 3.52-3.49 (m, 4H), 3.38-3.36 (m, 2H), 3.14 (s, 3H), 2.86 (d, J = 12.8 Hz, 2H), 2.35 (s, 2H), 2.24-2.20 (m, 2H).

LCMS M/z 608.1 $(M+H)^+$. HRMS anal. calcd for $C_{29}H_{36}F_2N_3O_7S$: 608.2242 $[M+H]^+$,

found

608.223.

Compound 1:

2-(2-{[(1,3-Benzothiazol-2-yl)methyl]carbamoyl}-2,3-dihydro-1H-inden-2-yl)acetic acid

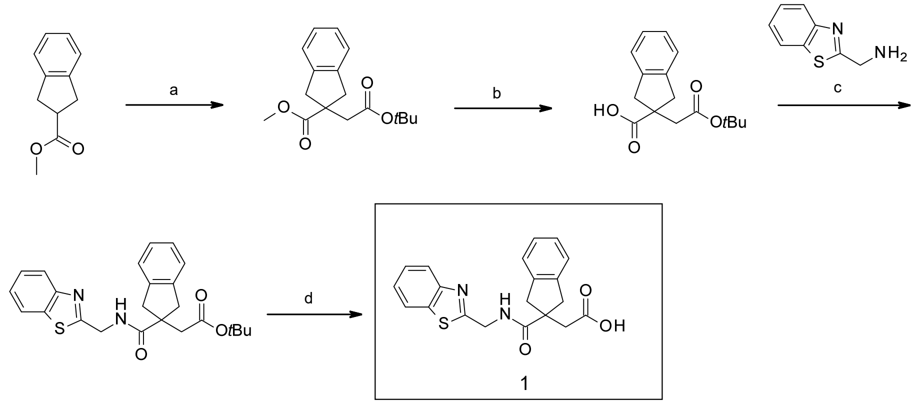

Fig. S4; Synthesis of Compound 1

a. Br-CH_2-CO_2tBu, NaHMDS b. KOH, EtOH/H_2O c. HATU, DIPEA d. TFA, DCM

i. Methyl 2-[2-(*tert*-butoxy)-2-oxoethyl]-2,3-dihydro-1H-indene-2-carboxylate

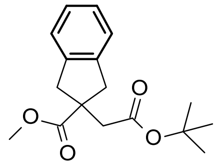

A solution of commercially available methyl 2,3-dihydro-1H-indene-2-carboxylate (4.53 g, 25.7 mmol) in THF (100 mL) was treated with a solution of sodium hexamethylsilazide in THF (1M, 38.6 ml, 38.8 mmol) dropwise at -70 °C under N_2. After 0.25h, *tert*-butyl bromoacetate (5.7 mL, 38.6 mmol) was added dropwise, ensuring the internal temperature did not rise above -60°C. After the addition was complete the mixture was allowed to warm to RT over 0.5h then quenched with saturated aq. ammonium chloride solution (150 mL). The mixture was extracted three times with EtOAc and the combined organic extracts were washed with brine, dried ($MgSO_4$) and evaporated (11.2 g). This was chromatographed on silica eluting with 0-40% EtOAc in hexane affording methyl 2-[2-(*tert*-butoxy)-2-oxoethyl]-2,3-dihydro-1H-indene-2-carboxylate as a yellow oil (5.7 g, 77%).
LCMS M/z 291.4 $(M+H)^+$ and M/z 235.3 $(M+H)^+$ (loss of *iso*butene)

ii. 2-[2-(*tert*-Butoxy)-2-oxoethyl]-2,3-dihydro-1H-indene-2-carboxylic acid

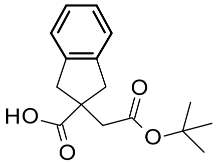

A solution of methyl 2-[2-(*tert*-butoxy)-2-oxoethyl]-2,3-dihydro-1H-indene-2-carboxylate (4.0 g, 13.8 mmol) in EtOH/H_2O (80 mL/40 mL) was treated with KOH (4.3 g, 75.8 mmol). After 3.5h the mixture was diluted with water and washed with ether. The aqueous phase was acidified to pH3 with solid citric acid monohydrate and extracted with EtOAc. The combined organic extracts were washed with brine, dried (Na_2SO_4) and evaporated affording the crude product as a solid (3.7 g). This was dissolved in chloroform, filtered and evaporated to give 2-[2-(*tert*-butoxy)-2-oxoethyl]-2,3-dihydro-1H-indene-2-carboxylic acid as a white solid (1.76 g, 47%).

LCMS M/z 277.3 $(M+H)^+$.

iii. *tert*-Butyl 2-(2-{[(1,3-benzothiazol-2-yl)methyl]carbamoyl}-2,3-dihydro-1H-inden-2-yl)acetate

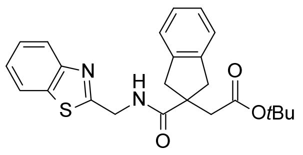

A solution of 2-[2-(*tert*-butoxy)-2-oxoethyl]-2,3-dihydro-1H-indene-2-carboxylic acid (530 mg, 1.92 mmol), HATU (876 mg, 2.3 mmol) and DIPEA (1.34 mL, 7.7 mmol) in DMF (10 mL) was stirred for 0.25h then 1,3-benzothiazol-2-ylmethanamine (350 mg, 1.74 mmol) was added. The mixture was stirred for 2.5 days then evaporated. The residue was dissolved in EtOAc and washed with saturated aq. $NaHCO_3$ solution, water and brine, then the organic extract was dried (Na_2SO_4) and evaporated affording the crude product (628 mg). This was chromatographed on silica eluting with 0-100% EtOAc in hexane affording *tert*-butyl 2-(2-{[(1,3-benzothiazol-2-yl)methyl]carbamoyl}-2,3-dihydro-1H-inden-2-yl)acetate as a white solid (520 mg, 64%).
LCMS M/z 423.2 $(M+H)^+$.

iv. 2-(2-{[(1,3-Benzothiazol-2-yl)methyl]carbamoyl}-2,3-dihydro-1H-inden-2-yl)acetic acid

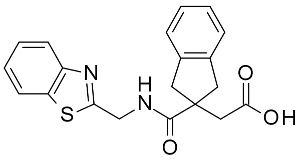

A solution of *tert*-butyl 2-(2-{[(1,3-benzothiazol-2-yl)methyl]carbamoyl}-2,3-dihydro-1H-inden-2-yl)acetate (520 mg, 1.23 mmol) in DCM (20 mL) was treated with TFA (2 mL). After 0.75h a further portion of TFA (1 mL) was added. The mixture was stirred overnight then evaporated, azeotroping twice with toluene. The residue was chromatographed on silica eluting with 0-100% EtOAc in hexane affording an oil which was dissolved in ACN/water and freeze dried affording the title compound as a white solid (327 mg, 72%).
^{1}H NMR (d6-DMSO) δ 8.80-8.70 (m, 1H), 8.05 (m, 1H), 7.92 (m, 1H), 7.50 (m, 1H), 7.40 (m, 1H), 7.20 (m, 2H), 7.15 (m, 2H), 4.67 (d, 2H), 3.45 (d, *J* = 16 Hz, 2H), 3.00 (d, *J* = 16 Hz, 2H), 2.75 (2H, s).

LCMS M/z 367.4 $(M+H)^+$. HRMS anal. calcd for $C_{20}H_{19}N_2O_3S$: 367.1116 $[M+H]^+$, found

367.1105.

Compound 2:

2-[2-[(5-chloro-1,3-benzothiazol-2-yl)methylcarbamoyl]indan-2-yl]acetic acid

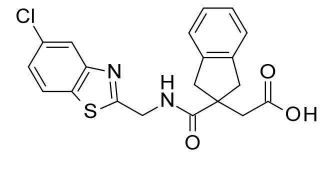

This was prepared in a similar manner to Compound 1, except using 5-chloro-1,3-benzothiazol-2-ylmethanamine.

^{1}H NMR (d6-DMSO) δ 8.90-8.80 (bs, 1H), 8.10 (m, 1H), 8.00 (s, 1H), 7.45 (m, 1H), 7.25 (m, 2H), 7.15 (m, 2H), 4.68 (m, 2H), 3.45 (d, J = 16 Hz, 2H), 3.00 (d, J = 16 Hz, 2H), 2.74 (s, 2H).

LCMS M/z 401.8 $(M+H)^+$ and M/z 403.7 $(M+H)^+$. HRMS anal. calcd for $C_{20}H_{18}ClN_2O_3S$: 401.0727 $[M+H]^+$, found 401.0717.

Compound 3:

2-[2-[(5-methoxy-1,3-benzothiazol-2-yl)methylcarbamoyl]indan-2-yl]acetic acid

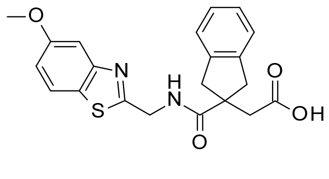

This was prepared in a similar manner to Compound 1, except using 5-methoxy-1,3-benzothiazol-2-ylmethanamine.

^{1}H NMR (d6-DMSO) δ 8.85-8.75 (bs, 1H), 7.90 (m, 1H), 7.45 (s, 1H), 7.25 (m, 2H), 7.15 (m, 2H), 7.05 (m, 1H), 4.65 (m, 2H), 3.85 (s, 3H), 3.45 (d, J = 16 Hz, 2H), 3.00 (d, J = 16 Hz, 2H), 2.72 (s, 2H).
LCMS M/z 397.5 $(M+H)^+$.

Compound 4:

2-[2-[(5,6-dimethoxy-1,3-benzothiazol-2-yl)methylcarbamoyl]indan-2-yl]acetic acid

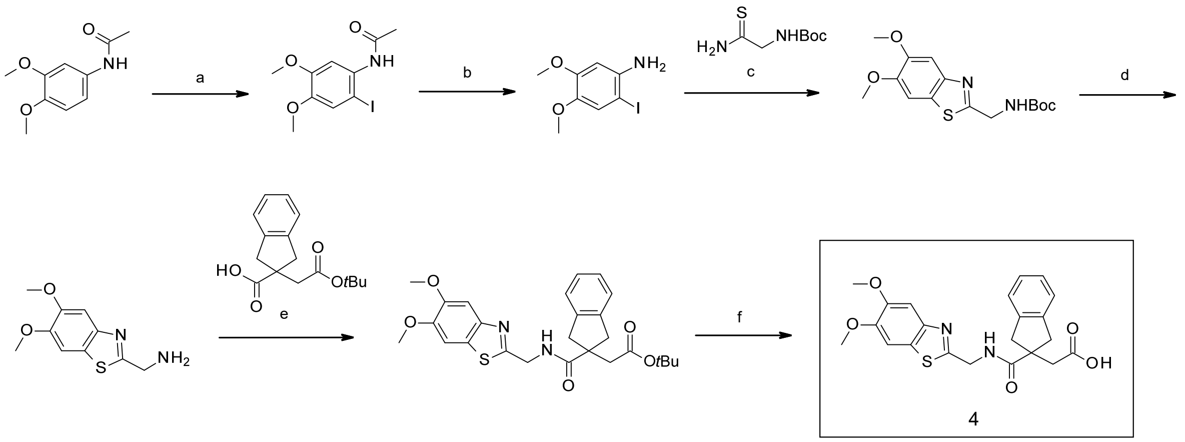

Fig. S5; Synthesis of Compound 4

a.ICl, AcOH b. NaOH, EtOH/H_2O c. $Pd_2(dba)_3$, dppf, CuO d. HCl, dioxane e. EDC.HCl, HOBt, TEA f. TFA, DCM

i. N-(2-iodo-4,5-dimethoxyphenyl)acetamide

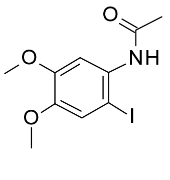

To a stirred solution of N-(3,4-dimethoxyphenyl)acetamide (12 g, 61.5 mmol) in DCM (600 mL) was added AcOH (12 mL) at RT and stirred for 10 min. Then iodine monochloride (12 g, 73.8 mmol) in DCM (100 mL) was added dropwise for 15 min. The dark brown solution was stirred for 4h at RT. The reaction mixture was diluted with DCM (200 mL), washed with water (2x100 mL), sat. sodium thiosulfate (2x100 mL) and brine. The organic extracts were then dried with Na_2SO_4, filtered and the solvent removed. The crude compound was purified by

chromatography (120 g silica cartridge, gradient 40% - 50% EtOAc/petroleum ether) to afford a pale yellow solid (5.2 g, 27%).

LCMS M/z 321.9 $(M+H)^+$.

ii. 2-iodo-4,5-dimethoxyaniline

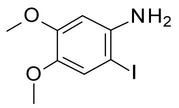

To a solution of N-(2-iodo-4,5-dimethoxyphenyl)acetamide (5.2 g, 16.1 mmol) in EtOH:H_2O (2:1, 150 mL) was added sodium hydroxide (32.3 g, 810 mmol) at RT. The reaction mixture was stirred at 100°C for 6h. The reaction mixture was concentrated under reduced pressure to give the residue. The residue was partitioned between EtOAc and cold water and the phases were separated. The organic extracts were washed with brine, dried with Na_2SO_4, filtered and the solvent removed. The crude compound was triturated with n-pentane and Et_2O to yield the product (4.1 g, 91%) as a light brown sticky solid.

LCMS M/z 280 $(M+H)^+$.

iii. *Tert*-butyl ((5,6-dimethoxybenzo[d]thiazol-2-yl)methyl)carbamate

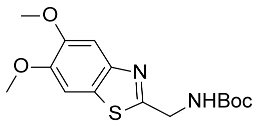

To a stirred solution of 2-iodo-4,5-dimethoxyaniline (1 g, 3.5 mmol) in DMF (10 mL) was added *tert*-butyl (2-amino-2-thioxoethyl)carbamate (1 g, 5.3 mmol), CuO (425 mg, 5.3 mmol) and purged with argon for 10 min. Then $Pd_2(dba)_3$ (164 mg, 0.17 mmol) and dppf (198 mg, 0.35 mmol) was added and then reaction mixture was purged with argon for 5 min. The mixture was stirred at 110°C in sealed tube for 16h. The reaction mixture was allowed to cool to RT, diluted with EtOAc (50 mL) and water (50 mL). It was filtered through a celite pad, washing with EtOAc (100 mL). The organic layer was separated, washed with water, brine, dried with

Na_2SO_4, filtered and the solvent removed by evaporation. The residue was purified by chromatography (24 g silica cartridge, gradient 50% - 75% EtOAc/petroleum ether) to afford a light brown solid (400 mg, 21%).
LCMS M/z 325.1 $(M+H)^+$.

iv. (5,6-dimethoxybenzo[d]thiazol-2-yl)methanamine hydrochloride

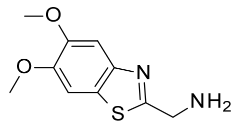

To a solution of *tert*-butyl ((5,6-dimethoxybenzo[d]thiazol-2-yl)methyl)carbamate (900 mg, 2.7 mmol) in 1,4-dioxane (5 mL) was added 4M HCl in dioxane (5 mL) at RT. The reaction mixture was stirred at RT for 4h. The reaction mixture was concentrated under reduced pressure. The resulting residue was triturated with n-pentane and Et_2O to afford a pale brown solid (700 mg).
LCMS M/z 225.1 $(M+H)^+$.

v. *Tert*-butyl 2-(2-(((5,6-dimethoxybenzo[d]thiazol-2-yl)methyl)carbamoyl)-2,3-dihydro-1H-inden-2-yl)acetate

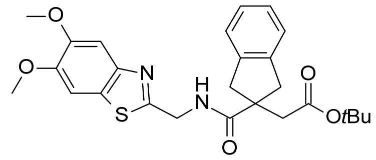

To a suspension of (5,6-dimethoxybenzo[d]thiazol-2-yl)methanamine hydrochloride (700 mg, 2.6 mmol) in DMF (10 mL) was added Et_3N (817 mg, 8.0 mmol) at RT and stirred for 10 min. Then 2-(2-(*tert*-butoxy)-2-oxoethyl)-2,3-dihydro-1H-indene-2-carboxylic acid (817 mg, 2.9 mmol), EDC.HCl (771 mg, 4.0 mmol) and HOBt (363 mg, 2.6 mmol) were added and the reaction mixture was stirred at RT for 16 h. It was diluted with water (50 mL) and stirred for 10 min. The resulting precipitate was filtered, washed with water and dried in vacuum. The residue was purified by chromatography (24 g silica cartridge, gradient 40% - 50% EtOAc/petroleum ether) to afford a yellow solid (510 mg, 40%).

LCMS M/z 483.2 $(M+H)^+$.

vi. 2-[2-[(5,6-dimethoxy-1,3-benzothiazol-2-yl)methylcarbamoyl]indan-2-yl]acetic acid

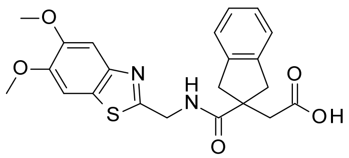

To a solution of *tert*-butyl 2-(2-(((5,6-dimethoxybenzo[d]thiazol-2-yl)methyl)carbamoyl)-2,3-dihydro-1H-inden-2-yl)acetate (500 mg, 1.07 mmol) in DCM (10 mL) was added TFA (5 mL) at 0°C. The reaction mixture was stirred vigorously at RT for 8h and the solvent was removed. The residue was triturated with n-pentane and Et_2O. The crude compound was purified by preparative HPLC [SYMMETRY-C8 (300*19), 7 u, Mobile phase: A: 0.1% FA in H_2O, B: ACN, Gradient: (T%B): 0/35, 8/80, 8.1/98, 10/98, 10.1/35, 13/35; Flow rate: 20 mL/min] to obtain an off-white solid (270 mg, 61%).

^{1}H NMR (500 MHz, DMSO-d_6): δ 12.13 (bs, 1H), 8.68 (t, J = 6.0 Hz, 1H), 7.55 (s, 1H), 7.44 (s, 1H), 7.22-7.20 (m, 2H), 7.15-7.13 (m, 2H), 4.60 (d, J = 6.0 Hz, 2H), 3.82 (s, 3H), 3.80 (s, 3H), 3.45 (d, J = 16.5 Hz, 2H), 2.98 (d, J = 16.5 Hz, 2H), 2.73 (s, 2H).

LCMS M/z 427 $(M+H)^+$. HRMS anal. calcd for $C_{22}H_{23}N_2O_5S$: 427.1327 $[M+H]^+$, found 427.1316.

Compound 5:

2-[2-([1,3]dioxolo[4,5-f][1,3]benzothiazol-6-ylmethylcarbamoyl)indan-2-yl]acetic acid

i. N-(benzo[d][1,3]dioxol-5-yl)acetamide

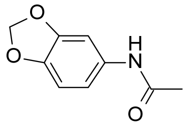

To a stirred solution of benzo[d][1,3]dioxol-5-amine (4.5 g, 32.8 mmol) in AcOH (70 mL) was added acetic anhydride (35 mL) at RT and stirred at RT for 16h. The reaction mixture was slowly poured into saturated aqueous $NaHCO_3$ solution and stirred for 10 min. The mixture

was extracted with DCM (2x100 mL). The organic layer was washed with water (2x100 mL) and brine. The organic extracts were then dried with Na_2SO_4, filtered and the solvent removed. The crude material was purified by chromatography (40 g silica cartridge, gradient 50% - 60% EtOAc/petroleum ether) to yield the product (4.6 g, 79%) as a pale brown solid.
LCMS M/z 180 $(M+H)^+$.

ii. 2-[2-([1,3]dioxolo[4,5-f][1,3]benzothiazol-6-ylmethylcarbamoyl)indan-2-yl]acetic acid

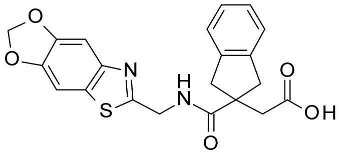

The title product was synthesized following the same synthetic sequence as described for the synthesis of compound 4. It was purified by preparative HPLC [SYMMETRY-C8 (300*19), 7 u, Mobile phase: A: 0.1% FA in H_2O, B: ACN, Gradient: (T%B):- 0/35, 8/75, 8.1/98, 10/98, 10.1/35, 13/35; Flow rate: 20 mL/min], yield 69%, affording a white solid (25 mg).
^1H NMR (500 MHz, DMSO-d_6): δ 12.13 (bs, 1H), 8.71 (bs, 1H), 7.54 (s, 1H), 7.42 (s, 1H), 7.22-7.19 (m, 2H), 7.15-7.12 (m, 2H), 6.10 (s, 2H), 4.59 (d, J = 6 Hz, 2H), 3.44 (d, J = 16.5 Hz, 2H), 2.98 (d, J = 16.5 Hz, 2H), 2.72 (s, 2H).

LCMS M/z 411.1 $(M+H)^+$. HRMS anal. calcd for $C_{21}H_{19}N_2O_5S$: 411.1015 $[M+H]^+$, found 411.1000.

Compound 6:

2-[2-[[6-(4-methylpiperazin-1-yl)-1,3-benzothiazol-2-yl]methylcarbamoyl]indan-2-yl]acetic acid

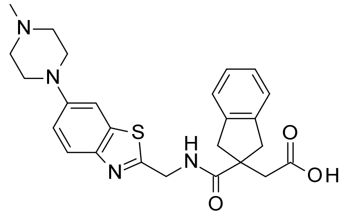

N-methylpiperazine (40.5 mg, 0.40 mmol) and K_3PO_4 (171.6 mg, 1.01 mmol) were added to a stirred solution of 2-[2-[(6-bromo-1,3-benzothiazol-2-yl)methylcarbamoyl]indan-2-yl]acetic acid (120 mg, 0.26 mmol) in THF (5 mL) at RT and the reaction mixture was purged with argon for 15 min. Ruphos Pd G1 (39.2 mg, 0.05 mmol) was added. The reaction mixture was purged with argon for further 5 min, stirred in a sealed tube at 80°C for 20h then filtered through celite pad, washing with EtOAc (50 mL). The filtrate was concentrated under reduced pressure. The crude material was purified by preparative HPLC (YMC-TRIART-C18 (150*30); 10 u; Flow rate: 25 mL/min; mobile phase: 0.05 % FA in H_2O: ACN) affording the title compound as a yellow solid (23 mg, 18%).

^{1}H NMR (d6-DMSO) δ 10.27 (bs, 1H), 8.39 (bs, 1H), 7.71 (d, *J* = 9.0 Hz, 1H), 7.46 (d, *J* = 2.0 Hz, 1H), 7.19-7.11 (m, 5H), 4.59 (d, *J* = 4.5 Hz, 2H), 3.43 (d, *J* = 16.0 Hz, 2H), 3.18-3.16 (m, 4H), 2.95 (d, *J* = 16.5 Hz, 2H), 2.57 (bs, 2H), 2.47-2.45 (m, 4H), 2.22 (s, 3H).

LCMS M/z 465.2 $(M+H)^+$. HRMS anal. calcd for $C_{25}H_{29}N_4O_3S$: 465.1960, found 465.1948 $[M+H]^+$.

Compound 7:

2-[2-[[5-[2-(dimethylamino)ethoxy]-1,3-benzothiazol-2-yl]methylcarbamoyl]indan-2-yl]acetic acid

i. *Tert*-butyl N-[(5-bromo-1,3-benzothiazol-2-yl)methyl]carbamate

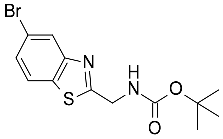

To a stirred solution of 5-bromo-2-iodo-aniline (3.0 g, 10.13 mmol) and *tert*-butyl (2-amino-2-thioxoethyl) carbamate (1.92 g, 10.13 mmol) in DMF (30 mL) was added CuO (0.8 g, 10.13

mmol) at RT and the reaction mixture was degassed with argon for 15 minutes. Then dppf (280 mg, 0.50 mmol) and $Pd_2(dba)_3$ (185.4 mg, 0.20) were added and the resulting reaction mixture was degassed with argon for further 5 minutes. It was stirred in sealed tube at 60°C for 3h, and then filtered through a celite pad, and washing with EtOAc (50 mL). The filtrate was washed with water (2x30 mL) and concentrated under reduced pressure. The crude compound was purified by chromatography on silica eluting with 20% EtOAc in petroleum ether, affording a yellow solid (5.0 g, 72%).
LCMS M/z 343 $(M+H)^+$.

ii. *Tert*-butyl N-[[5-(4,4,5,5-tetramethyl-1,3,2-dioxaborolan-2-yl)-1,3-benzothiazol-2-yl]methyl]carbamate

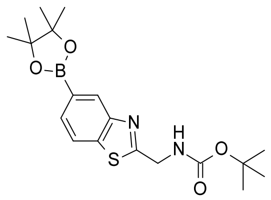

To a stirred solution of *tert*-butyl N-[(5-bromo-1,3-benzothiazol-2-yl)methyl]carbamate (1.3 g, 3.80 mmol), and BPin (1.44 g, 5.70 mmol) in 1,4-dioxane (15 mL) was added KOAc (745 mg, 7.60 mmol) at RT and the reaction mixture was purged with argon for 15 minutes. Then $PdCl_2$(dppf).DCM (155 mg, 0.190 mmol) was added and the reaction mixture purged with argon for further 5 minutes. It was heated to reflux in sealed tube for 12h, and then filtered through a celite pad, washing with EtOAc (50 mL). The filtrate was washed with water (2 x 30 mL), then the organic layer was dried with sodium sulphate, filtered and concentrated under reduced pressure obtain a brown solid (1.5 g, 100%).

LCMS M/z 391.2 $(M+H)^+$.

iii. *Tert*-butyl N-[(5-hydroxy-1,3-benzothiazol-2-yl)methyl]carbamate

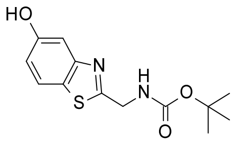

To a stirred solution of *tert*-butyl N-[[5-(4,4,5,5-tetramethyl-1,3,2-dioxaborolan-2-yl)-1,3-benzothiazol-2-yl]methyl]carbamate (1.5 g, 3.84 mmol) in THF (15 mL) was added 1N NaOH (3.84 mL g, 3.84 mmol) at 0°C and stirred for 10 minutes. Then H_2O_2 (30% in H_2O, 0.21 mL, 8.84 mmol) was added at 0°C and the reaction mixture stirred at RT for 1h. The reaction mixture was partitioned between EtOAc (100 mL) and water (70 mL). The aqueous phase was extracted with EtOAc (2 x 100 mL) and the combined organic extracts were washed with brine, dried with Na_2SO_4, filtered and evaporated. The residue was purified by chromatography on silica, eluting with 40% EtOAc in petroleum ether affording a white solid (1.0 g, 93% over 2 steps).
LCMS M/z 281.1 $(M+H)^+$.

iv. *tert*-butyl N-[[5-[2-(dimethylamino)ethoxy]-1,3-benzothiazol-2-yl]methyl]carbamate

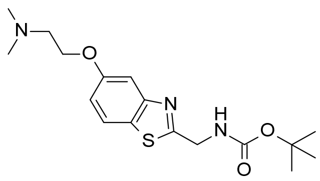

To a stirred solution of *tert*-butyl ((5-hydroxybenzo[d]thiazol-2-yl)methyl)carbamate (300 mg, 1.07 mmol) in ACN (5 mL) was added K_2CO_3, (221 mg, 1.60 mmol) and 2-bromo-N,N-dimethylethanamine hydrobromide (298 mg, 1.28 mmol) at RT. The reaction mixture was stirred in sealed tube at 90°C for 2h, then partitioned between EtOAc (80 mL) and water (40 mL). The aqueous phase was extracted with EtOAc (2 x 30 mL) and the combined organic extracts were washed with brine, dried Na_2SO_4, filtered and evaporated. The residue was purified by chromatography on silica eluting with 40%-50% EtOAc/petroleum ether to afford a pale yellow solid (344 mg, 92%).
LCMS M/z 510.1 $(M+H)^+$.

v. 2-[2-[[5-[2-(dimethylamino)ethoxy]-1,3-benzothiazol-2-yl]methylcarbamoyl]indan-2-yl]acetic acid

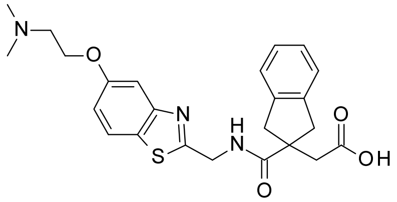

The title product was prepared from *tert*-butyl N-[[5-[2-(dimethylamino)ethoxy]-1,3-benzothiazol-2-yl]methyl]carbamate (60 mg) by the same TFA deprotection protocol as for Compound 16. The compound was purified by preparative HPLC [YMC-TRIART–C18-(150*25 mm), 10 u, mobile phase: A: 0.05 % FA in H_2O, B: ACN, Gradient (%B): 0/5, 8/50, 8.1/98, 10/98, 10.1/5, 12/5, Flow rate: 25 mL/min], affording a white solid (31 mg, 58%). ^{1}H NMR (500 MHz, DMSO-d_6); δ 12.2 (bs, 1H), 8.80 (bs, 1H), 7.87 (d, J = 8.5 Hz, 1H), 7.47 (d, J = 2.5 Hz, 1H), 7.22-7.20 (m, 2H), 7.15-7.13 (m, 2H), 7.03 (dd, J = 8.5 Hz, J = 2.5 Hz, 1H), 4.63 (d, J = 6 Hz, 2H), 4.12 (t, J = 6 Hz, 2H), 3.48 (d, J = 16.5 Hz, 2H), 3.01 (d, J = 16.5 Hz, 2H), 2.73 (s, 2H), 2.68 (t, J = 6 Hz, 2H), 2.26 (s, 6H).

LCMS M/z 454.1 $(M+H)^+$. HRMS anal. calcd for $C_{24}H_{28}N_3O_4S$ 454.1801$[M+H]^+$, found 454.1843.

Compound 8:

2-[2-[[6-(4,4-dimethylpiperazin-4-ium-1-yl)-1,3-benzothiazol-2-yl]methylcarbamoyl]indan-2-yl]acetate

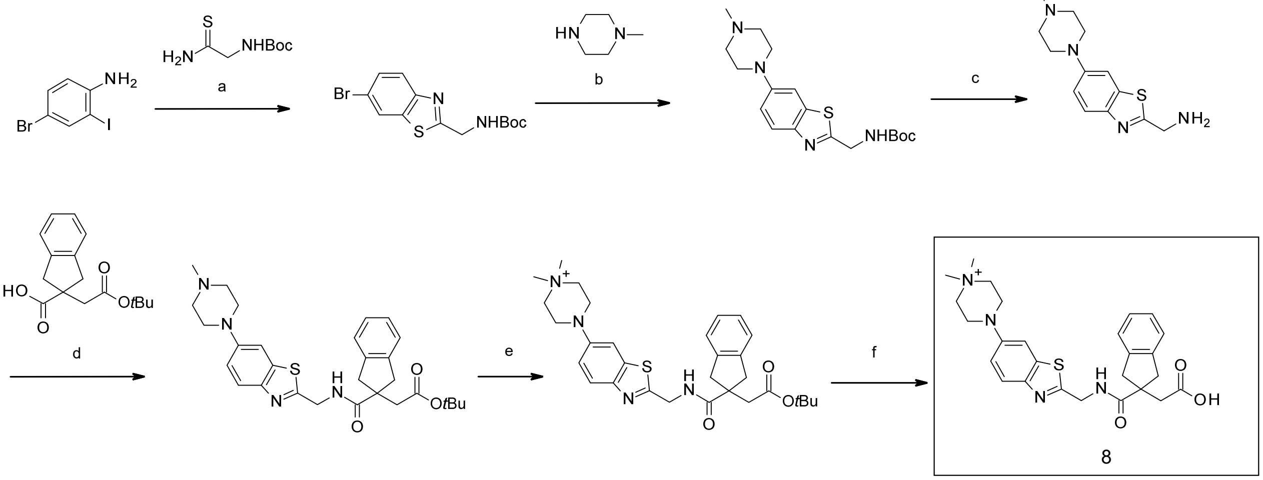

Fig. S6, Synthesis of Compound 8

a. $Pd_2(dba)_3$, dppf, CuO b. RuPhos Pd G1, K_3PO_4 c. HCl, dioxane. d. T_3P TEA e. MeI, THF, RT. f. TFA, TES

i. ***tert*-butyl N-[(6-bromo-1,3-benzothiazol-2-yl)methyl]carbamate**

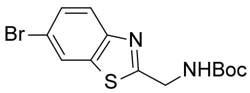

CuO (0.8 g, 10.13 mmol) was added to a stirred solution of 4-bromo-2-iodo-aniline (3 g, 10.13 mmol) and *tert*-butyl N-(2-amino-2-thioxo-ethyl)carbamate (1.92 g, 10.13 mmol) in DMF (30 mL) at RT and the reaction mixture was degassed with argon for 15 min. Then dppf (280 mg, 0.50 mmol) and $Pd_2(dba)_3$ (185.4 mg, 0.20) were added to the reaction mixture and degassed with argon for further 5 min. The reaction mixture was stirred in sealed tube at 60°C for 3h and filtered through a celite pad, washing with EtOAc (50 mL). The filtrate was washed with water (2x30 mL) and concentrated under reduced pressure. The crude compound was purified by silica gel chromatography, eluting with 20% EtOAc in petroleum ether affording a yellow solid (5 g, 72%).
LCMS M/z 343.0 $(M+H)^+$.

ii. *tert*-butyl N-[[6-(4-methylpiperazin-1-yl)-1,3-benzothiazol-2-yl]methyl]carbamate

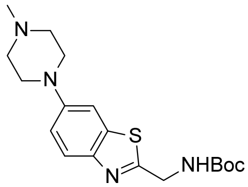

N-methyl piperazine (657.8 mg, 6.57 mmol) and K_3PO_4 (2.8 g, 13.1 mmol) were added to a stirred solution of *tert*-butyl N-[(6-bromo-1,3-benzothiazol-2-yl)methyl]carbamate (1.5 g, 4.38 mmol) in THF (20 mL) at RT and the reaction mixture was purged with argon for 15 min. Ruphos Pd G1 (639.1 mg, 0.87 mmol) was added. The reaction mixture was then purged with argon for further 5 min, stirred in a sealed tube at 80°C for 16h and filtered through celite pad, washing with EtOAc (50 mL). The filtrate was concentrated under reduced pressure. The crude compound was purified by chromatography on silica eluting with 4% MeOH in DCM affording a yellow solid (550 mg, 34%).
LCMS M/z 363.2 $(M+H)^+$.

iii. [6-(4-methylpiperazin-1-yl)-1,3-benzothiazol-2-yl]methanamine hydrochloride

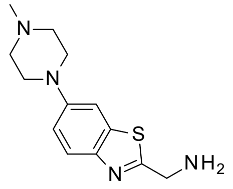

4N HCl in dioxane (4 mL) was added to a solution of *tert*-butyl N-[[6-(4-methylpiperazin-1-yl)-1,3-benzothiazol-2-yl]methyl]carbamate (550 mg, 1.51 mmol) in dioxane (5 mL) at 0°C. The reaction mixture was stirred at RT for 4h and concentrated under reduced pressure. The crude compound was triturated with n-pentane (5 mL) and Et_2O (5 mL) affording a white solid (450 mg, 100%).
LCMS M/z 263.1 $(M+H)^+$.

iv. *tert*-butyl 2-[2-[[6-(4-methylpiperazin-1-yl)-1,3-benzothiazol-2-yl]methylcarbamoyl]indan-2-yl]acetate

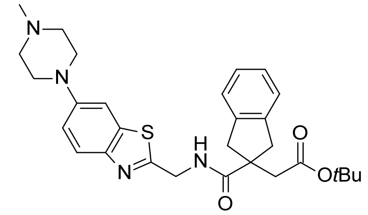

Et_3N (1.1 mL, 8.05 mmol) was added to a stirred solution of [6-(4-methylpiperazin-1-yl)-1,3-benzothiazol-2-yl]methanamine hydrochloride (400 mg, 1.34 mmol) in DMF (5 mL) at RT and stirred for 15 min. 2-(2-*tert*-butoxy-2-oxo-ethyl)indane-2-carboxylic acid (407 mg, 1.47 mmol) and T_3P (1.18 mL, 2.01 mmol) were then added. The reaction mixture was stirred at RT for 16h, diluted with water (30 mL) and extracted with EtOAc (2x50 mL). The combined organic layer was washed with brine, dried over Na_2SO_4, filtered and concentrated under reduced pressure. The crude compound was purified by chromatography on silica eluting with 4% MeOH in DCM affording a yellow solid (160 mg, 23% over 3 steps).
LCMS M/z 521.3 $(M+H)^+$.

v. *tert*-butyl 2-[2-[[6-(4,4-dimethylpiperazin-4-ium-1-yl)-1,3-benzothiazol-2-yl]methylcarbamoyl]indan-2-yl]acetate iodide

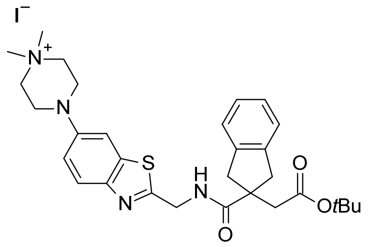

MeI (130 mg, 0.92 mmol) was added to a solution of *tert*-butyl 2-[2-[[6-(4-methylpiperazin-1-yl)-1,3-benzothiazol-2-yl]methylcarbamoyl]indan-2-yl]acetate (160 mg, 0.30 mmol) in THF (3 mL) at 0°C. The reaction mixture was stirred at RT for 4h and concentrated under reduced pressure. The crude residue was triturated with Et_2O (2x10 mL) affording a yellow solid which was used as such in the next step (160 mg).
LCMS M/z 535.2 $(M)^+$.

vi. 2-[2-[[6-(4,4-dimethylpiperazin-4-ium-1-yl)-1,3-benzothiazol-2-yl]methylcarbamoyl]indan-2-yl]acetic acid

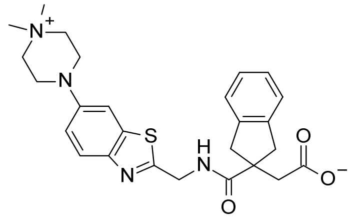

TFA:TES (5:1, 2 mL) was added to a stirred solution of *tert*-butyl 2-[2-[[6-(4,4-dimethylpiperazin-4-ium-1-yl)-1,3-benzothiazol-2-yl]methylcarbamoyl]indan-2-yl]acetate (120 mg, 0.22 mmol) in DCM (5 mL) at RT. The reaction mixture was stirred at RT for 2h and concentrated under reduced pressure. The residue was washed with n-pentane (10 mL) and Et_2O (10 mL). The crude material was purified by preparative HPLC (X-BRIDGE-C18 (150*30); 5 u; Flow rate: 25 mL/min; mobile phase: 0.05 % FA in H_2O: ACN) affording the title product as a pale pink solid (52 mg, 49%).
^{1}H NMR (d6-DMSO) δ 12.50 (bs, 1H), 7.80 (d, *J* = 9.0 Hz, 1H), 7.62 (d, *J* = 2.5 Hz, 1H), 7.23 (dd, *J* = 9.0 Hz, *J* = 2.5 Hz, 1H), 7.19-7.14 (m, 2H), 7.12-7.09 (m, 2H), 4.61 (d, *J* = 5.5 Hz, 2H), 3.57-3.55 (m, 8H), 3.39 (d, *J* = 16 Hz, 2H), 3.18 (s, 6H), 2.89 (d, *J* = 16 Hz, 2H), 2.38 (s, 2H).

LCMS M/z 479.3 $(M+H)^+$. HRMS anal. calcd for $C_{26}H_{30}N_4O_3S$: 479.2117 $[M+H]^+$, found 479.2111.

Compound 9:

2-[2-[[5-[2-(trimethylammonio)ethoxy]-1,3-benzothiazol-2-yl]methylcarbamoyl]indan-2-yl]acetate

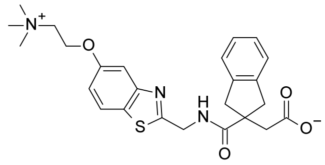

The title product was synthesised from *tert*-butyl ((5-hydroxybenzo[d]thiazol-2-yl)methyl)carbamate following the same procedure described for the synthesis of Compound 12. The crude material was purified by preparative HPLC [SUNFIRE-C18 (150*30 mm), 5 u, mobile phase: A: 0.05 % FA in H_2O, B: ACN, Gradient (%B): 0/10, 7/40, 8/40, 8.1/98, 9/98, 9.1/10, 12/10, Flow rate: 25 mL/min], affording a white solid (35 mg, 54%).

^{1}H NMR (500 MHz, DMSO-d_6): δ 11.72 (bs, 1H), 7.95 (d, J = 9 Hz, 1H), 7.59 (d, J = 2.5 Hz, 1H), 7.17-7.15 (m, 2H), 7.12-7.07 (m, 3H), 4.66 (d, J = 5 Hz, 2H), 4.56 (bs, 2H), 3.81 (t, J = 5 Hz, 2H), 3.40 (d, J = 16 Hz, 2H), 3.18 (s, 9H), 2.91 (d, J = 16 Hz, 2H), 2.47 (s, 2H).

LCMS M/z 468.2 $(M)^+$. HRMS anal. calcd for $C_{25}H_{30}N_3O_4S$ $(M+H)^+$: 468.1951, found 468.1944.

Compound 10:

2-(2-((benzo[d]thiazol-2-ylmethyl)carbamoyl)-5,6-difluoro-2,3-dihydro-1H-inden-2-yl)acetic acid

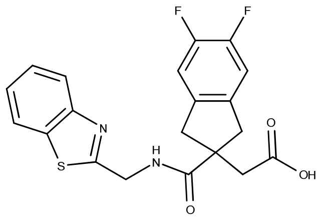

i. **2-(2-((benzo[d]thiazol-2-ylmethyl)carbamoyl)-5,6-difluoro-2,3-dihydro-1H-inden-2-yl)acetic acid**

This was synthesised using the method described for Compound 16 but using 1,3-benzothiazol-2-ylmethanamine. The title compound was isolated as a white solid (15 mg). ^{1}H NMR (d6-DMSO) δ 12.20 (bs, 1H), 8.99 (bs, 1H), 8.02 (d, *J* = 8.0 Hz, 1H), 7.92 (d, *J* = 8.0 Hz, 1H), 7.94 (dt, *J* = 7.7, 1.3 Hz, 1H), 7.40 (dt, *J* = 7.6, 1.1 Hz, 1H,), 7.28 (t, *J* = 9.2 Hz, 2H), 4.65 (*d*, *J* = 5.7 Hz, 2H), 3.43 (d, *J* = 16.4 Hz, 2H,), 2.99 (*d*, *J* = 16.4 Hz, 2H,), 2.76 (s, 2H).

LCMS M/z 425 $(M+Na)^+$. HRMS anal. calcd for $C_{20}H_{17}F_2N_2O_3S$ 403.0928 $[M+H]^+$, found 403.0916.

Compound 11:

2-[2-[[5-[3-(dimethylamino)propoxy]-6-methoxy-1,3-benzothiazol-2-yl]methylcarbamoyl]indan-2-yl]acetic acid

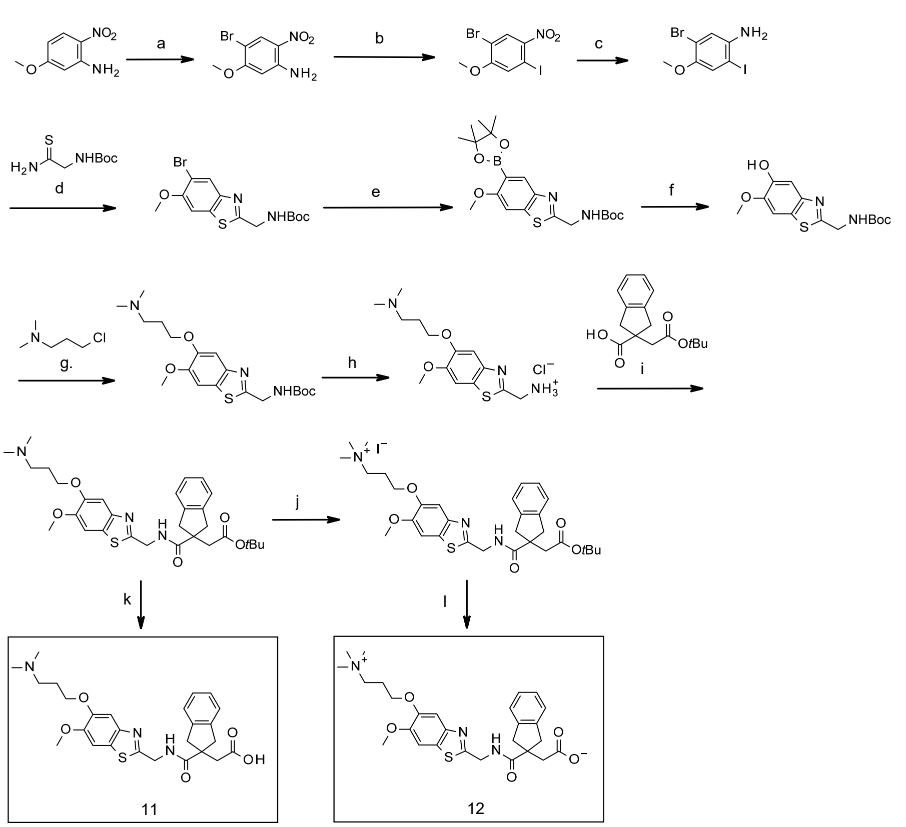

Fig. S7, Synthesis of Compounds 11 and 12

a. NBS, TFA, DCM. b. H_2SO_4, $NaNO_2$, KI, ACN:H_2O c. Fe, NH_4Cl, EtOH:H_2O d. $Pd_2(dba)_3$, dppf, CuO, acetonitrile. e. $PdCl_2$dppf, BPin, KOAc, DCM f. NaOH, H_2O_2, THF g. K_2CO_3, DMF, h. HCl, dioxane i. EDC.HCl, HOBt, TEA, DMF j. MeI, ACN k. and l. TFA, DCM

i. 4-bromo-5-methoxy-2-nitroaniline

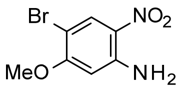

To a stirred solution of 5-methoxy-2-nitroaniline (100 g, 595 mmol) in ACN (2.5 L) 15 was added NBS (106 g, 595 mmol) portion wise at RT. The mixture was cooled to 0°C and treated with TFA (46 mL, 595 mmol) dropwise for 0.5h and allowed to stir at room temperature for 16h. The reaction mixture was diluted with water (1 L) and adjusted to ~ pH8 with 1N NaOH. The resulting precipitate was filtered, washed with water (500 mL) and dried under vacuum affording a yellow solid. (105 g, 72%). LCMS M/z 247 $(M+H)^+$.

ii. 1-Bromo-4-iodo-2-methoxy-5-nitrobenzene

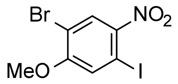

To a stirred solution of 4-bromo-5-methoxy-2-nitroaniline (50 g, 203 mmol) in ACN (750 mL) was added concentrated H2SO4 (24 mL, 457 mmol) dropwise at -10 °C. NaNO2 (28 g, 406 mmol) in water (175 mL) was added dropwise at -10 °C for 15 minutes and stirred at same temperature for 30 min. After that KI solution (135 g, 813 mmol) in water (175 mL) was added dropwise at -10 °C for 20 minutes and stirred at same temperature for 30 min. The reaction mixture was quenched with sodium metabisulphite solution (309 g, 1.62 mmol) in water (1.6 L) at -10 to 0°C for 1h. Then more water (1 L) was added and The mixture was stirred at RT for 0.5h. The resulting precipitate was filtered, washed with water (1 L) and dried under vacuum affording a yellow solid (60 g, 82%).
LCMS M/z 357.8 $(M+H)^+$.

iii. 5-Bromo-2-iodo-4-methoxyaniline

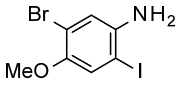

To a stirred solution of 1-bromo-4-iodo-2-methoxy-5-nitrobenzene (106 g, 296 mmol) in EtOH: H2O (800 mL: 200 mL) was added Fe (49.7 g, 890 mmol), NH4Cl (80 g, 1.48 mmol) at RT and the mixture was heated at 90°C for 2h. It was then cooled to 60°C, treated with additional amounts of Fe (33 g, 593 mmol) and NH4Cl (80 g 1.48 mmol) then stirred at 90°C for 0.5h. The reaction mixture was filtered through a celite pad, washing with methanol (1 L) and the filtrate was concentrated. The residue was treated with cold water (1 L) and adjusted to ~pH8 with 1N NaOH. The resulting precipitate was filtered and dried *in vacuo* affording a light brown solid (90 g, 92%).
LCMS M/z 327.8 $(M+H)^+$.

iv. *Tert*-butyl N-[(5-bromo-6-methoxy-1,3-benzothiazol-2-yl)methyl]carbamate

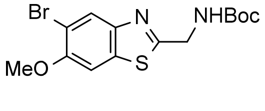

To a stirred solution of 5-bromo-2-iodo-4-methoxyaniline (50 g, 152 mmol) in ACN (560 mL) was added *tert*-butyl(2-amino-2-thioxoethyl)carbamate (35 g, 183 mmol), CuO (17 g, 305 mmol) and the mixture was degassed with argon for 20 minutes. Pd2(dba)3 (14 g, 15.2 mmol) and dppf (25.4 g, 15.8 mmol) were added and the mixture purged with argon for further 5 minutes before the reaction mixture was heated at 80°C for 4h. The mixture was filtered through a celite pad, washing the pad with EtOAc (300 mL). The filtrate was washed with water and evaporated to obtain a solid. This was dissolved in ACN (200 mL) and after 1h the mixture was filtered, washing with ACN (50 mL) and dried *in vacuo* affording an off white solid (34 g, 60%).
LCMS M/z 372.9 $(M+H)^+$.

v. *Tert*-butyl N-[[6-methoxy-5-(4,4,5,5-tetramethyl-1,3,2-dioxaborolan-2-yl)-1,3-benzothiazol-2-yl]methyl]carbamate

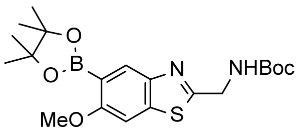

To a stirred solution of *tert*-butyl ((5-bromo-6-methoxybenzo[d]thiazol-2-yl)methyl)carbamate (5 g, 13.44 mmol) in dioxane (100 mL) was added BPin (6.8 g, 26.8 mmol) and KOAc (4.6 g, 47.0 mmol), followed by degassing with argon for 0.25h. Then Pd2Cl2(dppf) (1.1 g, 1.34 mmol) was added and purged again with argon for further 5 minutes. The reaction mixture was heated at 100°C for 16h then filtered through a celite pad, washing with EtOAc (50 mL). The filtrate was washed with water, brine and evaporated affording a white solid (12 g).
LCMS M/z 339 $(M+H)^+$.

vi. *Tert*-butyl N-[(5-hydroxy-6-methoxy-1,3-benzothiazol-2-yl)methyl]carbamate

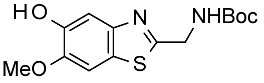

To a stirred solution of *tert*-butyl N-[[6-methoxy-5-(4,4,5,5-tetramethyl-1,3,2-dioxaborolan-

2-yl)-1,3-benzothiazol-2-yl]methyl]carbamate (12 g, 35.5 mmol) in THF (180 mL) was added 1N NaOH (35 mL, 35.5 mmol), 30% H2O2 (6.2 mL 81.6 mmol) at 0 °C and the mixture stirred for 0.5h at 0°C. It was partitioned between water and EtOAc. The organic layer was separated, washed with water, brine and evaporated. The residue was chromatographed on silica eluting with 30% EtOAc in petroleum ether affording a white solid (2.5 g 54% over 2 steps).
1H NMR (500 MHz, CDCl3): δ 7.50 (s, 1H), 7.25 (s, 1H), 5.76 (s, 1H), 5.30 (s, 1H), 4.68 (d, *J* = 10 5.5 Hz, 2H), 3.97 (s, 3H), 1.54 (s, 9H).
LCMS M/z 311.0 $(M+H)^+$.

vii. *Tert*-butyl N-[[5-[3-(dimethylamino)propoxy]-6-methoxy-1,3-benzothiazol-2-yl]methyl]carbamate

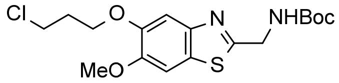

To a solution of *tert*-butyl N-[(5-hydroxy-6-methoxy-1,3-benzothiazol-2-yl)methyl]carbamate (750 mg, 2.41 mmol) in DMF (5 mL) was added K_2CO_3 (1 g, 7.25 mmol), 3-chloro-N,N-dimethylpropan-1-amine (355 mg, 2.90 mmol) at RT and the mixture was heated at 80°C for 4h. It was diluted with water (25 mL) and extracted with EtOAc (2 x 30 mL). The organic extract was dried, filtered and evaporated affording a brown oil (1.0 g).
LCMS M/z 395.8 $(M+H)^+$.

viii. 3-[[2-(aminomethyl)-6-methoxy-1,3-benzothiazol-5-yl]oxy]-N,N-dimethyl-propan-1-amine hydrochloride

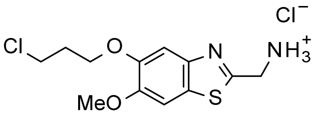

To a solution of *tert*-butyl N-[[5-[3-(dimethylamino)propoxy]-6-methoxy-1,3-benzothiazol-2-yl]methyl]carbamate (1.0 g, 2.53 mmol) in dioxane (5 mL) was added 4M HCl in dioxane (6mL) at RT and the mixture stirred for 6h. It was evaporated and the resulting residue was triturated with diethyl ether (25 mL) affording a pale yellow solid (0.92g).
LCMS M/z 296.2 $(M+H)^+$.

ix. *tert*-butyl 2-[2-[[5-[3-(dimethylamino)propoxy]-6-methoxy-1,3-benzothiazol-2-yl]methylcarbamoyl]indan-2-yl]acetate

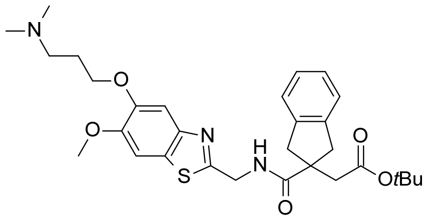

To a solution of 3-[[2-(aminomethyl)-6-methoxy-1,3-benzothiazol-5-yl]oxy]-N,N-dimethylpropan-1-amine hydrochloride (450 mg, 1.52 mmol) in DMF (6 mL) was added Et3N (1.1mL, 7.62 mmol) and stirred for 10 minutes. Then 2-(2-(*tert*-butoxy)-2-oxoethyl)-2,3-dihydro-1H-indene-2-carboxylic acid (463 mg, 1.67 mmol), EDC. HCl (440 mg, 2.28 mmol) and HOBt (210 mg, 1.52 mmol) were added at RT and stirred for 16h. The reaction mixture was diluted with water (30 mL) and extracted with EtOAc (2 x 40 mL) then evaporated. The residue was chromatographed on silica eluting with10-12% MeOH in DCM affording a yellow solid (310 mg, 56%).
LCMS M/z 554.2 $(M+H)^+$.

x. 2-[2-[[5-[3-(dimethylamino)propoxy]-6-methoxy-1,3-benzothiazol-2-yl]methylcarbamoyl]indan-2-yl]acetic acid

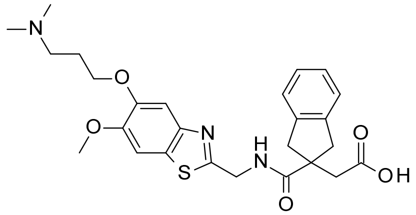

To a solution of *tert*-butyl 2-[2-[[5-[3-(dimethylamino)propoxy]-6-methoxy-1,3-benzothiazol-2-yl]methylcarbamoyl]indan-2-yl]acetate (120 mg, 0.21 mmol) in DCM (5 mL) was added TFA (2 mL) at 0°C and the mixture stirred at RT for 2h. It was evaporated and the residue was triturated with diethyl ether (15 mL). The crude compound was purified by preparative HPLC [YMC-TRIART(150 X 25 mm), 10 u, Mobile phase: A: 0.1% FA in H2O, B: ACN] affording the title compound as an off-white solid (32 mg, 30%).

^{1}H NMR (500 MHz, DMSO-d6): δ 9.00 (bs, 1H), 7.55 (s, 1H), 7.43 (s, 1H), 7.21-7.20 (m, 2H), 7.14-7.12 (m, 2H), 4.60 (d, *J* = 6 Hz, 2H), 4.04 (t, *J* = 6.5 Hz, 2H), 3.81 (s, 3H), 3.45 (d, *J* = 16 Hz, 2H), 2.98 (d, *J* = 16 Hz, 2H,), 2.69 (s, 2H), 2.40 (t, *J* = 7 Hz, 2H), 2.17 (s, 6H), 1.91-1.85 (m, 2H).

LCMS M/z 498.1 $(M+H)^+$. HRMS anal. calcd for $C_{26}H_{32}N_3O_5S$ 498.2063$[M+H]^+$, found 498.1944.

Compound 12:
2-[2-[[6-methoxy-5-[3-(trimethylammonio)propoxy]-1,3-benzothiazol-2-yl]methylcarbamoyl]indan-2-yl]acetate

i. **3-[[2-[[[2-(2-*tert*-butoxy-2-oxo-ethyl)indane-2-carbonyl]amino]methyl]-6-methoxy-1,3-benzothiazol-5-yl]oxy]propyl-trimethyl-ammonium iodide**

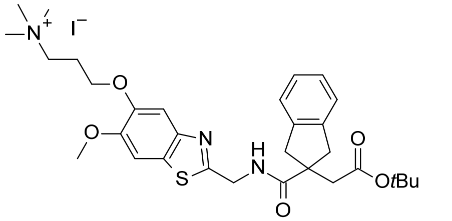

To a solution of *tert*-butyl 2-[2-[[5-[3-(dimethylamino)propoxy]-6-methoxy-1,3-benzothiazol-2-yl]methylcarbamoyl]indan-2-yl]acetate (200 mg, 0.36 mmol) in ACN (5 mL) was added MeI (1 mL) at 0°C and stirred at RT for 16h. The mixture was evaporated and residue was purified by chromatography on silica eluting with 15-20% 7M NH3/MeOH in DCM affording a yellow solid (100 mg, 49%).
LCMS M/z 568.3 $(M)^+$.

ii. **2-[2-[[6-methoxy-5-[3-(trimethylammonio)propoxy]-1,3-benzothiazol-2-yl]methylcarbamoyl]indan-2-yl]acetate**

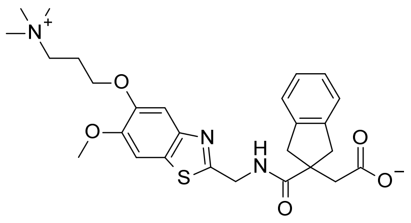

To a solution of 3-[[2-[[[2-(2-*tert*-butoxy-2-oxo-ethyl)indane-2-carbonyl]amino]methyl]-6-methoxy-1,3-benzothiazol-5-yl]oxy]propyl-trimethyl-ammonium iodide (90 mg, 0.15 mmol) in DCM (5 mL) was treated with TFA (1.5 mL) at 0°C and stirred at RT for 4h. The mixture was evaporated and the residue was triturated with diethyl ether (10 mL). The residue was purified by preparative HPLC [X-BRIDGE-C18 (150*30), 5 u, Mobile phase: A: 0.1% FA in H2O, B: ACN] affording the title compound as a white solid (8 mg, 10%).

^{1}H NMR (500 5 MHz, DMSO-d6): 12.27 (bs, 1H), 7.64 (s, 1H), 7.53 (s, 1H), 7.17-7.15 (m, 2H), 7.11-7.09 (m, 2H), 4.61 (d, *J* = 5.5 Hz, 2H), 4.12 (t, *J* = 6 Hz, 2H), 3.82 (s, 3H), 3.51-3.49 (m, 2H), 3.40 (d, *J* = 16 Hz, 2H), 3.10 (s, 9H), 2.90 (d, *J* = 16 Hz, 2H), 2.40 (s, 2H), 2.24-2.21 (m, 2H).

LCMS M/z 512.3 $(M+H)^+$. HRMS anal. calcd for $C_{27}H_{34}N_3O_5S$ $(M+H)^+$: 512.2219, found 512.2206.

Compound 13:

2-[2-[[5-[(1,1-dimethylpiperidin-1-ium-4-yl)methoxy]-6-methoxy-1,3-benzothiazol-2-yl]methylcarbamoyl]indan-2-yl]acetate

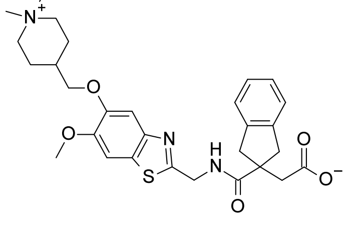

This was prepared from *tert*-butyl 2-[2-[[6-methoxy-5-[(1-methyl-4-piperidyl)methoxy]-1,3-benzothiazol-2-yl]methylcarbamoyl]indan-2-yl]acetate, which was accessed using the same

chemistry as for Compound 12 with the difference that 4-(chloromethyl)-1-methyl-piperidine was used in the phenol alkylation step. Quaternisation of *tert*-butyl 2-[2-[[6-methoxy-5-[(1-methyl-4-piperidyl)methoxy]-1,3-benzothiazol-2-yl]methylcarbamoyl]indan-2-yl]acetate with MeI followed by TFA deprotection protocol as described for Compound 16 afforded the title compound as a white solid (22 mg).

^{1}H NMR (500 MHz, DMSO-d$_6$): δ 11.32 (bs, 1H), 7.61 (s, 1H), 7.50 (s, 1H), 7.18-7.16 (m, 2H), 7.12-7.10 (m, 2H), 4.61 (d, *J* = 5.5 Hz,2H), 4.02 (d, *J* = 6.5 Hz, 2H), 3.82 (s, 3H), 3.45-3.33 (bs, 6H), 3.10 (s, 3H), 3.05 (s, 3H), 2.91 (d, *J* = 16 Hz, 2H), 2.55-2.45 (bs, 2H), 2.07-2.04 (m, 1H), 1.94-1.92 (m, 2H), 1.79-1.74 (m, 2H).

LCMS M/z 538.1 (M+H)$^+$. HRMS anal. calcd for $C_{29}H_{36}N_3O_5S$ (M+H)$^+$: 538.2376, found 538.2366.

Compound 14:

2-[2-[[5-[(1,1-dimethylpiperidin-1-ium-4-yl)methoxy]-6-methoxy-1,3-benzothiazol-2-yl]methylcarbamoyl]-5,6-difluoro-indan-2-yl]acetate

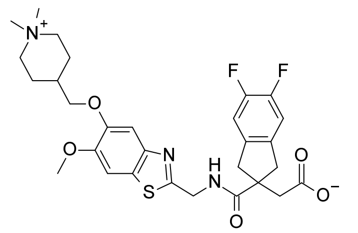

This was prepared from following the same procedure as for Compound 13, using 2-(2-(*tert*-butoxy)-2-oxoethyl)-5,6-difluoro-2,3-dihydro-1H-indene-2-carboxylic acid in the coupling step instead of 2-[2-(*tert*-butoxy)-2-oxoethyl]-2,3-dihydro-1H-indene-2-carboxylic acid, affording the compound as a white solid (56 mg).

^{1}H NMR (400 MHz, d$_6$-DMSO) δ 13.10 (bs 1H), 7.60 (s, 1H), 7.45 (s, 1H), 7.20 (t, 2H), 4.65 (d, 2H), 4.05 (m, 2H), 3.85 (s, 3H), 3.50 (m, 2H), 3.45 (d, 2H), 3.40 (m, 2H), 3.10 (s, 3H), 3.05 (s, 3H), 2.90 (d, 2H), 2.30 (s, 2H), 2.05 (m, 1H), 1.90 (m, 2H), 1.80 (m, 2H).

LCMS M/z 574.4 (M+H)$^+$. HRMS anal. calcd for $C_{29}H_{34}F_2N_3O_5S$ (M+H)$^+$: 574.2187, found 574.2174.

Compound 15:

2-[2-[[5-[3-[bis(2-hydroxyethyl)-methyl-ammonio]propoxy]-6-methoxy-1,3-benzothiazol-2-yl]methylcarbamoyl]indan-2-yl]acetate

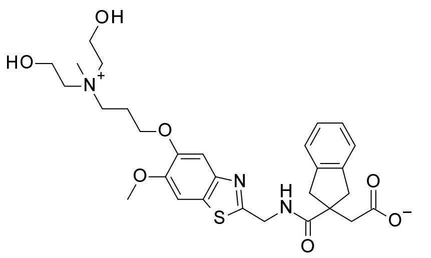

This was prepared in the same manner as Compound 16 but with the intermediate acid (1ii) as described for Compound 1. The title compound was isolated as a white solid (31 mg). ^{1}H NMR (400 MHz, d_6-DMSO) δ 13.00 (bs, 1H), 7.65 (s, 1H), 7.50 (s, 1H), 7.20 (m, 2H), 7.10 (m, 2H), 5.50 (bs, 2H), 4.60 (m, 2H), 4.20 (m, 2H), 3.95 (m, 4H), 3.85 (s, 3H), 3.60 (m, 2H), 3.50 (m, 4H), 3.40 (m, 2H), 3.15 (s, 3H), 2.90 (d, 2H), 2.20 (m, 2H).

LCMS M/z 572.4 $(M+H)^+$. HRMS anal. calcd for $C_{29}H_{37}N_3O_7S$: 572.243 $[M+H]^+$,

found

572.2417.

Protein crystallography

Data from the archived co-crystals was collected at the Diamond Light Source at Harwell, UK on beamline i04. A dataset to 2.7Å was obtained. Data were processed using Xia 3dii for integration and merged and truncated using AIMLESS. Structures were solved by molecular replacement using PHASER using the LasB phosphoramidon complex coordinates from the structure 3DBK as a search model. The structures were then manually refitted and refined using a combination of COOT and REFMAC from the CCP4 suite.

Data collection

X-ray Source DIAMOND I04 (IN20018_13)

Wavelength (Å) 0.97949

Space group P 1 21 1

Molecule/a.s.u

Cell dimensions 4

a, b, c (Å) 120.98 44.52 161.71

a, b, g (°) 90.00 100.61 90.00

Resolution (Å) 41.7-2.74

(2.81-2.74)*

Unique Reflections 44296 (3215)

Rmerge 0.351 (1.207)

I / sI 3.9 (1.1)

Completeness (%) 97.2 (96.4)

Redundancy 3.1 (3.0)

CC ½ 0.917 (0.305)

Refinement

Resolution (Å) 2.74

No. reflections 44290 (2143)

Rwork / Rfree   0.263/ 0.297

No. atoms

Protein   9354

Ligand/ion 168/12§

Water   28

B-factors

Protein   28.87

Ligand (s)   50.57/36.42

Water   21.53

R.m.s. deviations

Bond lengths (Å)   0.0143

Bond angles (°)   1.91

*Values in parentheses are for highest-resolution shell.

§ Number of atoms and B-factors for compounds (ligand of interest) and other heterogens present the structure are stated separately.

The data is publicly available in the PDB, reference 7QH1
